# Synergistic Parasite-Pathogen Interactions Mediated by Host Immunity Can Drive the Collapse of Honeybee Colonies

**DOI:** 10.1371/journal.ppat.1002735

**Published:** 2012-06-14

**Authors:** Francesco Nazzi, Sam P. Brown, Desiderato Annoscia, Fabio Del Piccolo, Gennaro Di Prisco, Paola Varricchio, Giorgio Della Vedova, Federica Cattonaro, Emilio Caprio, Francesco Pennacchio

**Affiliations:** 1 Dipartimento di Scienze Agrarie e Ambientali, Università degli Studi di Udine, Udine, Italy; 2 Centre for Immunity, Infection and Evolution, School of Biological Sciences, University of Edinburgh, Edinburgh, United Kingdom; 3 Dipartimento di Entomologia e Zoologia Agraria “Filippo Silvestri”, Università degli Studi di Napoli “Federico II”, Portici (Napoli), Italy; 4 Istituto di Genomica Applicata, Parco Scientifico e Tecnologico Luigi Danieli, Udine, Italy; Stanford University, United States of America

## Abstract

The health of the honeybee and, indirectly, global crop production are threatened by several biotic and abiotic factors, which play a poorly defined role in the induction of widespread colony losses. Recent descriptive studies suggest that colony losses are often related to the interaction between pathogens and other stress factors, including parasites. Through an integrated analysis of the population and molecular changes associated with the collapse of honeybee colonies infested by the parasitic mite *Varroa destructor*, we show that this parasite can de-stabilise the within-host dynamics of Deformed wing virus (DWV), transforming a cryptic and vertically transmitted virus into a rapidly replicating killer, which attains lethal levels late in the season. The de-stabilisation of DWV infection is associated with an immunosuppression syndrome, characterized by a strong down-regulation of the transcription factor NF-κB. The centrality of NF-κB in host responses to a range of environmental challenges suggests that this transcription factor can act as a common currency underlying colony collapse that may be triggered by different causes. Our results offer an integrated account for the multifactorial origin of honeybee losses and a new framework for assessing, and possibly mitigating, the impact of environmental challenges on honeybee health.

## Author Summary

Honeybees are of capital importance for humans since crop production significantly depends upon pollination by these insects. In recent years, widespread collapses of honeybee colonies have been reported throughout the world; unfortunately, despite intense research efforts, the causal agents of such losses are not yet identified, although parasites seem to play a key-role. We combined molecular, field-longitudinal and theoretical approaches to describe the mechanistic basis and dynamical properties of collapse-causing interactions within the multi-parasite community infecting the honeybees. We found that the parasitic mite *Varroa destructor* can de-stabilise the within-host dynamics of Deformed wing virus (DWV), transforming a cryptic and vertically transmitted virus into a rapidly replicating killer. The de-stabilisation of DWV infection results from a widespread immunosuppression characterized by a strong down-regulation of a member of the gene family NF-κB. This gene family not only plays a central role in insect immunity, but is also involved in intricate cross-talks with a number of physiological and stress response pathways. This suggests that different stress factors may alter the critical balance between viral pathogens and host-defences, promoting intense viral replication in bees harbouring silent infections and subsequent colony collapse. The model we propose can potentially explain the multifactorial origin of bee losses.

## Introduction

In the last few years, large-scale losses of honeybees (*Apis mellifera* L.) have been recorded all over the world [1]. A poorly understood syndrome, called Colony Collapse Disorder (CCD), reported in the United States of America since 2006 [2], has attracted the attention of both the scientific community and the public opinion [3], [4]. However, elevated winter colony losses, not related to CCD, have been reported in most regions of the northern hemisphere [5] and, even in the USA, CCD seems to be just one of the many causes of colony losses [6].

Several possible causes have been claimed for colony losses but there is now a general consensus about the fact that many factors are likely involved [7]. Whatever the origin, this problem has caused great concern due to the importance of honeybees as pollinators of many crops, which represent a significant and increasing proportion of human diet [8], [9]. Unfortunately, despite the considerable research efforts devoted to the study of the problem, the causes of widespread colony losses still remain poorly understood from a functional point of view, although pathogens seem to play a key-role [7], [10].

Several lines of direct and indirect evidence for the involvement of existing and emerging parasites and pathogens have been provided [11]–[17]. Recent studies suggest that, more generally, the collapse of honeybee colonies involves an interaction between pathogens and other stress factors, including the parasitic mite *Varroa destructor* Anderson & Trueman [2], [18], [19].

*V. destructor* is a widespread and economically important parasite of *A. mellifera* [20], [21], which can transmit pathogenic viruses, often associated with colony collapse [14], [16], [19], [22], [23], and determine a host immunosuppression syndrome not fully characterized at the molecular level [24]–[26]. Even though the possible role of the *Varroa* mite in colony losses is supported by a wealth of data [7], [17], [19], [27], and its active vectoring of bee viruses is demonstrated [28], the functional details of this dangerous association still remain poorly defined [21]. In particular, the association with Deformed wing virus (DWV) appears particularly interesting due to the increasing body of evidence about the role of this virus in bee colony losses [14]–[16], [19]. DWV is a positive strand RNA virus that can be vertically transmitted through the germ-line, causing covert infections in honeybee populations [29]. Available data suggest that DWV copy control can be undermined by concurrent infestation with *V. destructor*, leading to damaging overt infection [29]. However, although significant contributions have been provided [30]–[32], the mechanism of this interaction remains unclear.

Multi-parasite within-host interactions are receiving increasing attention [33] in order to achieve a better comprehension of the structure, dynamics and pathogenic significance of parasite communities [34]. Unfortunately, the descriptive nature of most studies carried out so far on honeybees has not allowed a detailed functional representation of the complex network of biotic interactions underpinning the decline of honeybee colonies.

The present study aims at filling this gap, by dissecting at the population and molecular level the major changes that underlie the colony collapse associated with *V. destructor* infestation, in order to describe both the mechanistic basis and dynamical properties of the biotic interactions that are involved. To address these issues we adopted an approach based on the comparative analysis of bee colonies exposed to different infestation levels of *V. destructor*, while maintained in the same environmental conditions. This allowed to accurately monitor the major changes occurring over time in the colonies and to shed light on the most crucial components involved in the decline and eventual collapse; laboratory experiments, carried out under strictly controlled conditions, complemented our field study.

The results allow us to define and analyse a novel dynamical model to describe the complex interactions between bees, pathogens and parasites and other stress factors, providing a new predictive framework for the study of the impact of diverse environmental stress factors on honeybee health.

## Results

### Dynamics of Bee Population

In an isolated location we set up two experimental apiaries, one of which received conventional acaricide treatments to control mite infestation (low infested colonies: LIC), while the other was left untreated, to monitor the effects of an increasing mite population (highly infested colonies: HIC).

**Figure 1:**
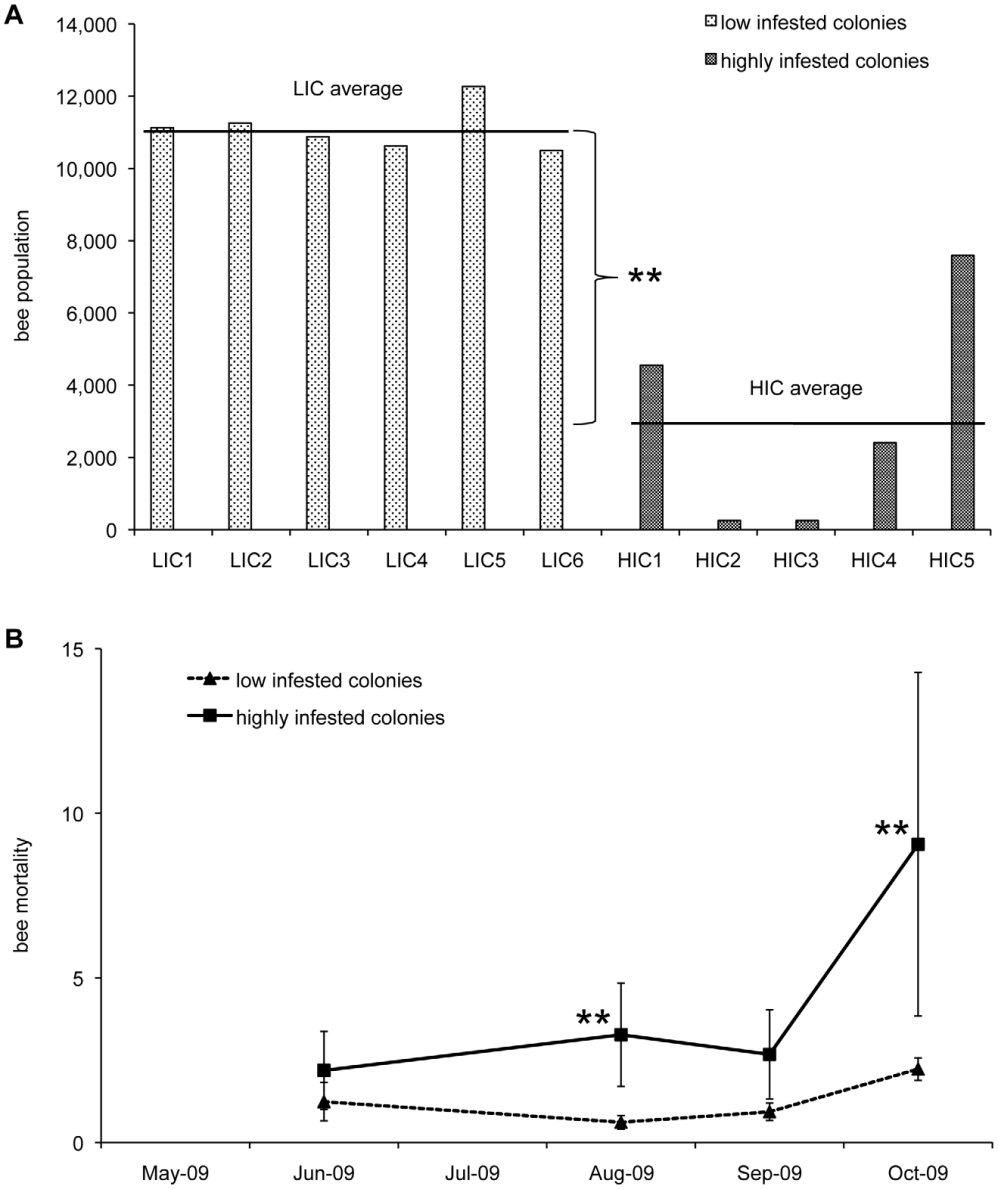
Seasonal dynamics of bees in colonies with low and high levels of mite infestation. (**A**) Estimated bee numbers recorded in each hive in October, when a sudden decrease of bee population was observed in highly infested colonies. (**B**) Bee mortality over time. The error bars indicate the standard deviation; mean values significantly different are denoted with asterisks (*P≤0.05; **P≤0.01). Bee population in highly infested colonies reached minimum levels in October, because of a marked increase of bee mortality.

A decline of bee population was observed in all colonies along the Summer, although a marked acceleration of the process was noted in HIC late in the season so that, at the end of October, a significant reduction of bee population was observed in such colonies (U = 0, n_1_ = 6, n_2_ = 5: *P*<0.01; Figure 1A). Two highly infested colonies collapsed by the end of Autumn, whereas the remaining ones did so by the following Spring. Bee mortality, as determined from the number of dead bees recovered in front of the hives and bee population, was abruptly and significantly raised at the end of the season in HIC (U = 0, n_1_ = 6, n_2_ = 5: *P*<0.01; Figure 1B).

### Parasites and Pathogens

**Figure 2:**
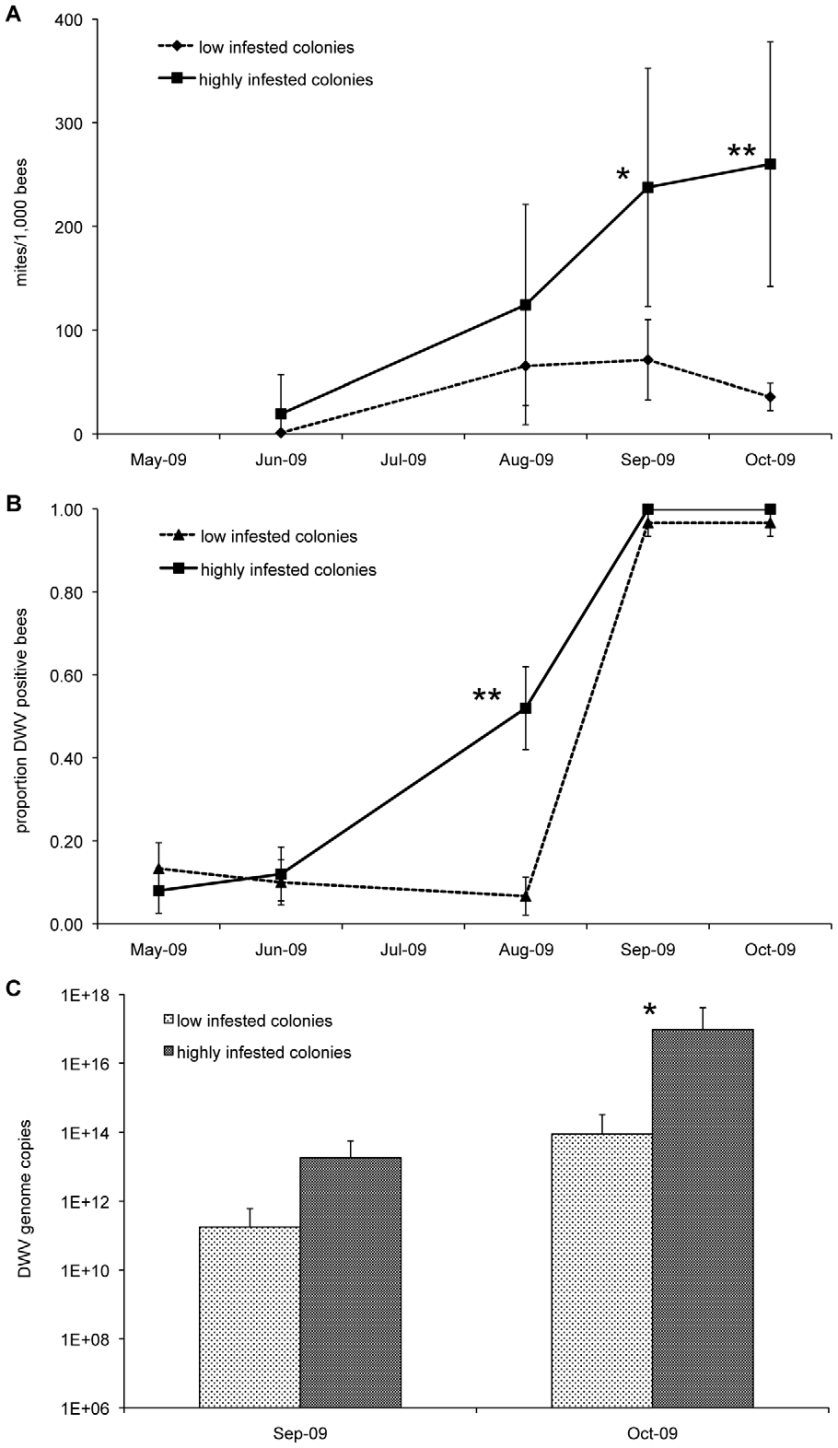
Mites and DWV in low and highly infested colonies. (**A**) Number of mites per 1,000 bees. (**B**) Seasonal prevalence of Deformed wing virus (DWV) in low and highly infested colonies. (**C**) Number of DWV genome copies in infected honeybees, collected in September and October from low and highly infested colonies. The error bars indicate the standard deviation; mean values significantly different are denoted with asterisks (*P≤0.05; **P≤0.01). Mite population steadily increased along the season in untreated colonies; DWV prevalence approached 100% at the end of the season both in low and highly infested colonies, but the number of genome copies was much higher in highly infested colonies.

After a steady increase over time, the HIC mite population reached its highest level at the end of the season, whereas acaricide treatments kept it under control in the LIC (Figure 2A).

A metagenomic analysis of bee samples collected in October from all experimental hives revealed the presence of a few common symbionts [11], [35] (Table S1). With regard to non-viral pathogens, *Nosema ceranae*, linked to colony losses in Spain [12], occurred at similar rates both in LIC and HIC (Table S1).

A survey by RT-PCR of the most common pathogenic bee viruses [22], carried out on bee samples collected monthly from the experimental hives, revealed the widespread presence of Black queen cell virus (BQCV), Deformed wing virus (DWV) and Sacbrood virus (SBV) only. Both BQCV and SBV prevalence fluctuated and overall declined along the season (Figure S1); in contrast, DWV prevalence increased over time and, in September, approached 100% in all experimental hives (Figure 2B), similar to reports in other studies [19], [36], [37].

Quantitative Real-Time RT-PCR analysis of DWV infected bees collected in October from the experimental hives showed that the number of viral genome copies was significantly higher in honeybees from HIC (U = 46, n_1_ = 14, n_2_ = 11: *P*≤0.05; Figure 2C), and that the significant increase of bee mortality recorded concurrently (Figure 1B) was associated with higher viral loads in infested colonies, exceeding 1×10^15^ genome copies per bee.

**Figure 3:**
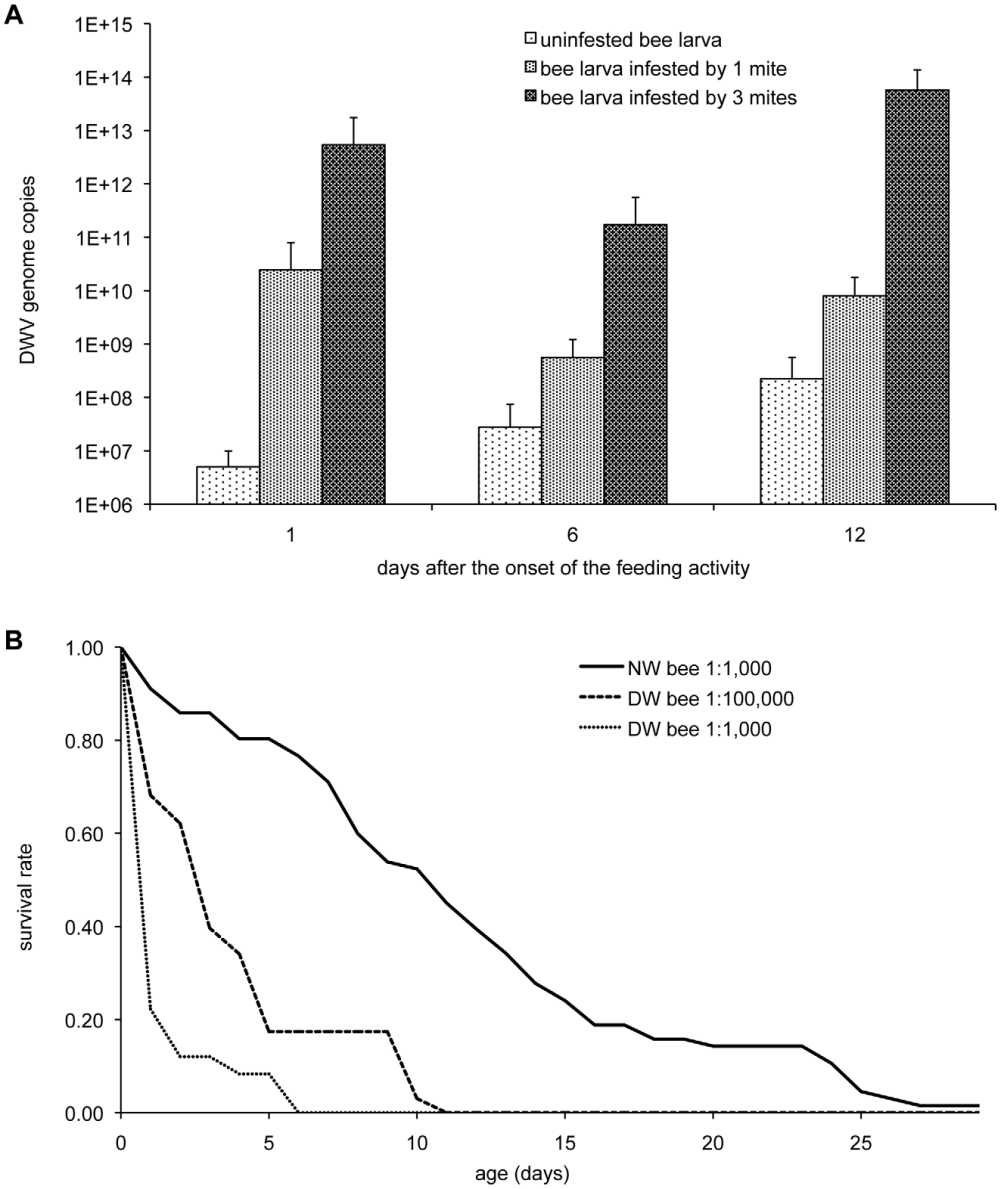
*Varroa* infestation and DWV genome copies in infested bees and the effect of viral load on bee survival. (**A**) Number of DWV genome copies in honeybees larvae artificially infested with different numbers of *V. destructor* mites, for different time intervals; the error bars indicate the standard error. (**B**) Survival of honeybees larvae injected with two different dilutions (1∶1,000 and 1∶100,000) of a whole body lysate of bees with deformed wings (DW) and of bees with normal wings as control (NW). Infestation by the *Varroa* mite caused increasing number of DWV genome copies in infected bees, this significantly affected bee longevity.

The increase of viral load associated with intense *Varroa* infestation and its lethal impact were further corroborated by laboratory experiments. A significant increase of DWV genome copies in artificially mite-infested honeybee larvae was triggered by *Varroa* feeding (H = 12.46, df = 2: *P*<0.01; Figure 3A). Moreover, the level of viral infection alone markedly influenced the survival of honeybees. In fact, injection of different dilutions of bee lysates obtained from individuals showing deformed wings resulted in rates of bee mortality significantly higher than in controls (M = 5.645 and 7.442 for lower and higher concentration respectively: *P*<0.001) and positively related to the lysate concentration used (M = 2.564: *P*<0.01; Figure 3B). This effect can be considered the result of different levels of DWV injected. In fact, this virus was exclusively present in the lysate of symptomatic bees, it was absent in control extracts, while both experimental lysates contained BQCV and were *Nosema*-free (data not shown).

### Honeybee Immune System

To shed light on the alterations of the honeybee immune system associated with *Varroa*-induced viral replication, we applied RNA-seq technology to perform a transcriptomic analysis of adult bees collected from each experimental colony in October, when the concurrent viral outbreak and the bee mortality peak were observed. An immunosuppressive effect was evident in bees collected from HIC, which was characterized by a significant down-regulation of 19 immune genes [38]. The most pronounced effects were observed on signalling molecules (e.g. *dorsal-1A*, a member of the protein family NF-κB, and serine proteases), while a relatively lower degree of down-regulation was recorded for those involved in recognition of non-self (e.g. AmSCR, scavenger receptors B5 and B7, C-type lectin 8) and for a few components of immune signalling pathways (e.g. Hem, Tak1, SOCS) (Table 1, Table S2). However, this immunosuppressive syndrome was associated with a significant up-regulation of 6 immune genes, encoding both recognition (PGRP-S2, NimC2, Eater-like) and signalling (serine proteases) molecules (Table 1, Table S2), part of them playing a role in phagocytosis. The differential expression of the genes which showed the most evident alteration of their transcriptional profile was confirmed by means of a Real-Time RT-PCR analysis of bee samples collected from the same colonies (Figure S2); in particular, the absolute quantification of *dorsal-1A*, the most down-regulated gene, with potential impact on several immune and stress responses, confirmed the strong reduction of transcript level observed late in the season in HIC (U = 2, n_1_ = 5, n_2_ = 5: *P*<0.05; Figure S3).

**Table 1:** Honeybee immune genes showing significant differences (*P*≤0.05) of their transcription level, as affected by different mite infestation densities.

Gene ID	Name	Family/pathway	RPKM (LIC) X±SD	RPKM (HIC) X±SD	Fold change
GB19066	dorsal-1A*	NF-κB/Toll	0.76±0.40	0.12±0.21	0.16
GB14309	cSP33*	serine proteases	0.13±0.09	0.03±0.04	0.22
GB13397	SPH51*	serine proteases	1.67±0.39	0.71±0.28	0.42
GB13813	AmSCR-B5	Scav. Receptor B	24.11±5.32	13.63±3.69	0.57
GB15549	AmSCR-B7	Scav. Receptor B	16.66±4.53	9.50±2.20	0.57
GB14642	IGFn3-1	IG Superfam. Genes	40.74±5.69	24.87±2.72	0.61
GB17018	Angiopoietin	Fibrinogen	4.90±1.18	3.07±0.32	0.63
GB18789	TEPA	TEP	9.41±0.88	6.05±1.45	0.64
GB17167	Hem	JNK	15.95±2.04	10.75±2.13	0.67
GB11846	IGFn3-7	IG Superfamily	24.27±1.47	16.78±0.72	0.69
GB11358	IGFn3-2	IG Superfamily	34.09±6.60	23.85±2.48	0.70
GB14382	CTL8	C-lectin domain	25.59±2.81	19.04±1.28	0.74
GB13522	MAPKKK9	MAPK	27.91±3.00	20.95±3.73	0.75
GB18949	SOCS	JakSTAT	89.91±10.51	67.66±5.18	0.75
GB14664	Tak1	IMD	32.92±3.47	24.94±2.29	0.76
GB11373	Rac	RAC1 protein	230.71±27.37	178±54±22.84	0.77
GB18324	Galectin-2	Galectin	21.50±1.74	17.18±1.48	0.80
GB10026	Galectin-1	Galectin	162.84±11.12	130.91±11.23	0.80
GB18923	STAT92E	JakSTAT	32.98±2.34	26.58±3.98	0.81
GB11320	RIP1	MAPK	14.08±1.83	19.37±3.71	1.38
GB14603	SP17	serine proteases	3500.37±541.87	4815.52±669.39	1.38
GB14654	SP11	serine proteases	5.99±1.80	11.65±3.18	1.95
GB14645	Eater-like*	EGF Family	14.64±4.84	33.27±13.01	2.27
GB13979	NimC2*	Phagocytosis	13.93±5.48	32.50±11.71	2.33
GB19301	PGRP-S2*	PGRP	226.29±52.86	981.11±868.53	4.34

The gene expression values, as Reads Per Kilobase of exon model per Million mapped reads (RPKM) [84], scored on bees from low infested and highly infested colonies are reported. The “fold change” represents the ratio between the average gene expression value of highly infested colonies and that of low infested ones; values smaller than one indicate a significant transcriptional down-regulation, while those higher than 1 indicate up-regulation. An asterisk marks genes whose differential expression was confirmed by Quantitative Real-Time RT-PCR (Figures S2 and S3). A significant down-regulation of several immune genes was observed in bees from highly infested colonies; the most marked effect was recorded for *dorsal-1A*, a member of the NF-κB gene family.

**Figure 4:**
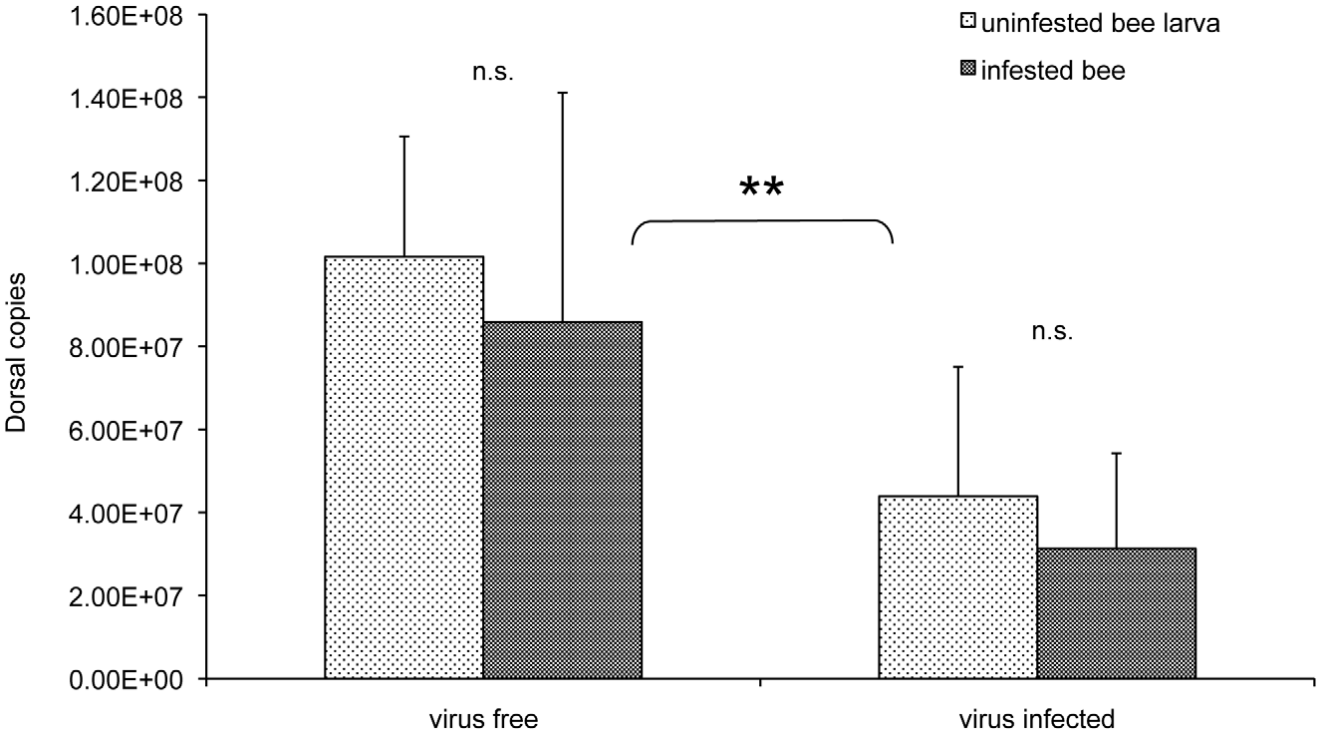
Dorsal expression in virus free and virus infected bees. Dorsal copies in virus free and virus infected honeybee larvae, either infested or not with one *Varroa* mite, 12 days after cell sealing; the error bars indicate the standard deviation. Average viral load in infected bee larvae, uninfested or infested by the *Varroa* mite, was 2.40E+10 and 3.22E+12, respectively. Dorsal expression was significantly reduced in virus infected bees compared to virus free bees, while *Varroa* infestation did not affect gene expression.

To tentatively assess the respective contribution of *Varroa* mites and DWV in the induction of the observed immunosuppression, we measured the transcriptional level of *dorsal-1A* in bees, either infected or not by DWV, as affected by infestation of *Varroa* mites *in vitro*. No significant differences in the level of the *dorsal-1A* transcript were induced by mite feeding in lab reared bees that resulted DWV-free at the end of the experiment; conversely the expression level of *dorsal-1A* in lab reared bees infected by DWV was significantly lower than in the case of virus-free individuals, irrespective of their exposure to mite infestation (F = 26.79, df = 1: *P*<0.001; Figure 4). This result indirectly indicates that the virus may play an important role in the observed transcriptional down-regulation of *dorsal-1A*, which could be considered part of the virulence strategy adopted by DWV to overcome one of the central components of the antiviral immunity in insects [39]–[45].

**Figure 5:**
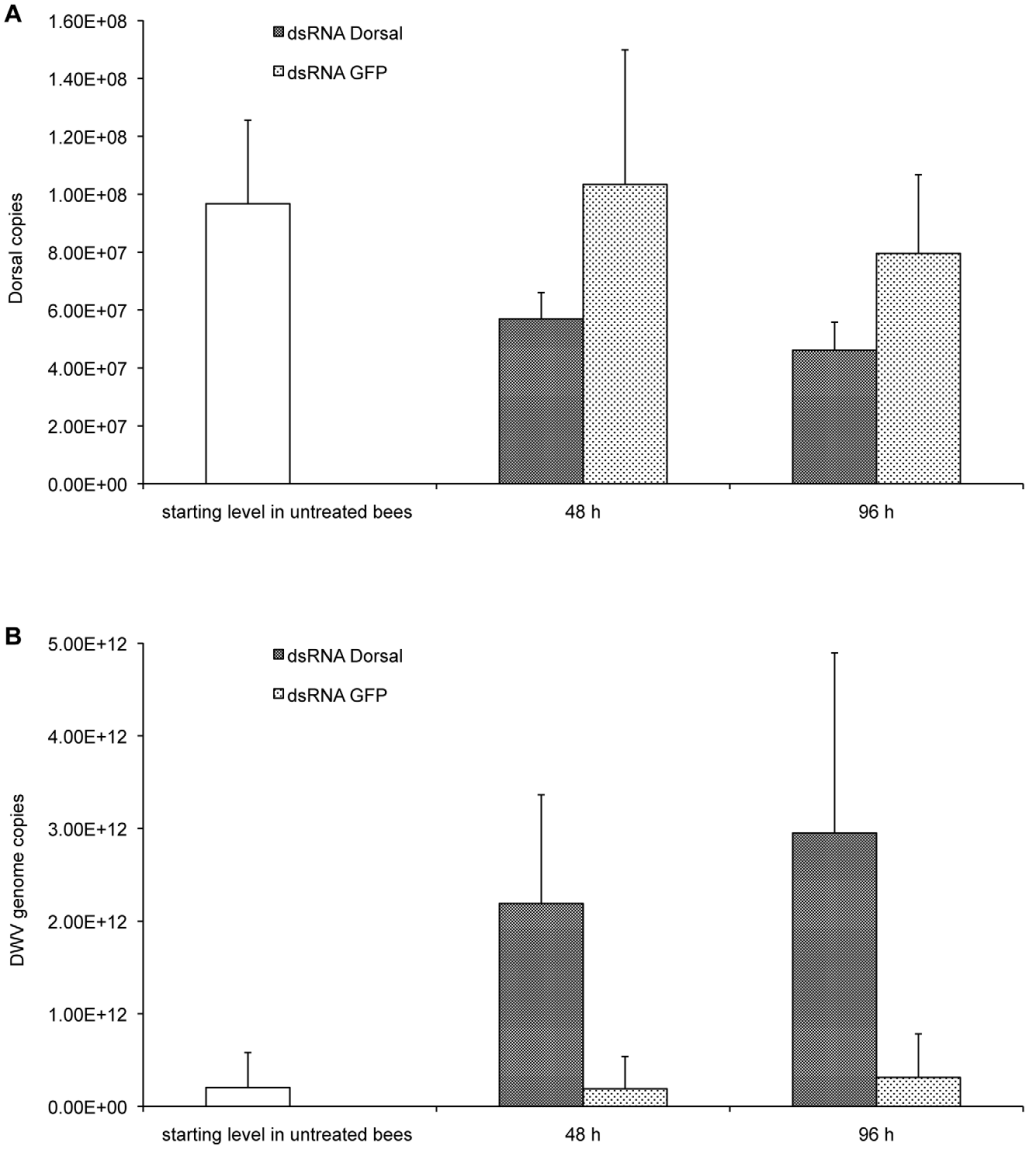
Effect of the down-regulation of the transcription factor *dorsal-1A* by RNAi on DWV replication in bees. (**A**) *Dorsal-1A* transcript level in bees fed for different times with a sucrose/protein solution, containing dsRNA of honeybee *dorsal-1A* (dsRNA Dorsal) or dsRNA of Green Fluorescent Protein (dsRNA GFP) as a control. (**B**) Deformed wing virus genome copies in bees treated as above. The error bars indicate the standard deviation. The significant rate (H = 7.00, df = 1: *P* = 0.008) of silencing of the target gene triggered a significant increase (H = 9.61, df = 1: *P* = 0.002) of viral replication.

To corroborate this hypothesis, we assessed whether *dorsal-1A* transcript abundance can affect viral infection in honeybees, by using RNA interference (RNAi) and measuring the resulting effects on viral load. We observed a significant suppression of *dorsal-1A* transcription in bees ingesting the corresponding dsRNA (H = 7.00, df = 1: *P* = 0.008; Figure 5A), along with a concurrent significant increase of DWV genome copies (H = 9.61, df = 1: *P* = 0.002; Figure 5B). This result demonstrates that a reduction of NF-κB availability promotes viral replication, and supports the hypothesis that this transcription factor is an important component of the antiviral response in honeybees. Moreover, it indirectly indicates that any stress factor triggering responses mediated by NF-κB can compete for the use of this transcription factor and promote viral replication.

### Dynamics of Multiple Interactions

**Figure 6:**
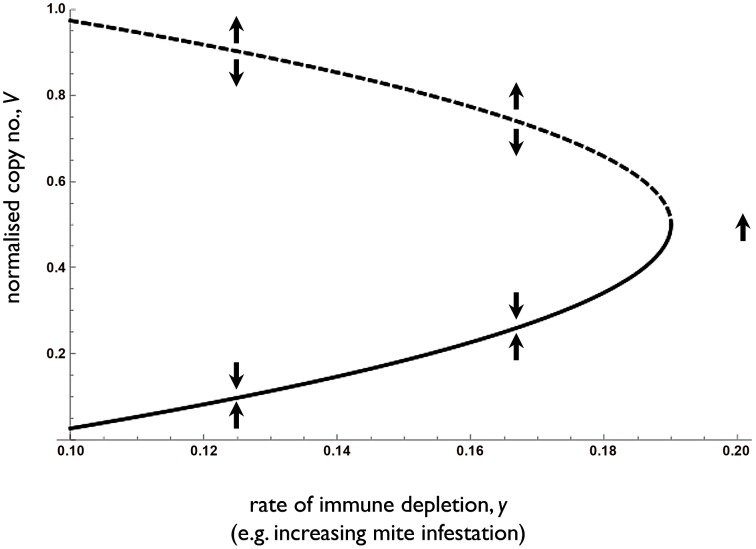
Accelerating or ‘threshold’ immuno-suppression by DWV can create bistable DWV dynamics. The stable (solid line) and unstable (dotted line) equilibrium level of DWV (arbitrary scale) are calculated from equations S4, S5, and plotted as a function of increasing levels of immune depletion (*y*). Below the dotted line, the virus can be efficiently regulated by the immune-system to some intermediate (potentially cryptic) density, represented by the solid line. Above the dotted line (and for high *y*, any point to right of intersection with solid line), the virus cannot be efficiently regulated and a viral explosion ensues. Any factor such as mite feeding that depletes the immune system (increasing *y*) will first cause a gradual increase in copy number, *V* (moving right along the solid line), and then at a defined point (intersection of solid and dotted lines), a viral explosion will ensue. Parameters are *x* = 0.09 (*y>x* ensures that the virus can invade from rare) and *z* = 0.4.

To explore the dynamical properties of our proposed pathogen-parasite interaction, we constructed and analysed a series of simple dynamical models of DWV copy number, mediated by a shared immune currency that can in turn be modified by the presence of virus and other stressors such as mite feeding. Under a default chronic infection model (with a constant rate of immuno-excitation) [46], we see a stable intermediate viral set-point. If, in contrast, we have constant immuno-suppression, then a purely aggressive viral dynamic results, with all successful infections leading to explosive growth. The simplest model consistent with the observed bistable copy number control (low, cryptic or high, overt infection) requires that the immunosuppressive effect of DWV displays some form of threshold function with increasing copy number. Given this assumption, Figure 6 and the corresponding analysis highlights that in the absence of any additional immunological strain on the host (such as mite feeding), DWV can be effectively regulated to low copy number, so long as DWV is kept below a high and critical threshold. However, any factor that depletes the immune system will cause a gradual increase in the stable set-point until a critical transition occurs and uncontrolled viral replication ensues. The sudden transition to explosive viral growth results directly from the non-linear immuno-suppressive behaviour of DWV, potentially allowing the virus to function as an opportunistic pathogen, sensing and exploiting host weakness with escalating immuno-suppression and explosive growth.

## Discussion

A steady decline of bee population during Summer, after the peak of nectar importation, is a common event under temperate climatic conditions, often followed by population collapse in untreated bee colonies exposed to increasing mite infestations [21]. However, the data reported here show that the decline of highly infested colonies is characterized by a sharp acceleration occurring at the end of the Summer.

The time course of mite infestation did not mirror the sudden increase in bee mortality, suggesting that other mortality factors, interacting with the *Varroa* mite, were likely involved but neither the metagenomic analysis nor the molecular survey of bee viruses revealed any relevant qualitative difference between HIC, that collapsed at the end of the season, and LIC, that survived in good condition. Instead, significant quantitative differences in DWV genome copies were found associated with different mite infestation levels, with bees from HIC bearing viral loads 10^3^ fold higher than bees exposed to a lower mite pressure, as reported also by other authors [19].

The field data were further corroborated by laboratory experiments showing that mite feeding triggers viral replication in bees, which show a mortality rate that is positively associated with the viral load.

The negative effects of the association between the *Varroa* mite and DWV have been widely investigated and many interesting details of this dangerous interaction have been revealed [19], [23], [24], [30]–[32], [47]–[50]. Experimental data on the impact of this interaction on honeybee health and colony stability are currently being expanded, largely on the basis of correlation studies, which allow a thorough analysis of the factors involved [14], [15], [19]. The present study builds upon this growing background information, by providing experimental evidence on the mechanistic details of this virus-mite association, trying to shed light on the functional link between *V. destructor* infestation, DWV abundance and bee mortality.

The transcriptomic analysis of adult bees, collected from each experimental colony in October, when viral replication rate was high, evidenced a severe alteration of the transcriptional profile of several immune genes. This immune syndrome was largely suppressive, with the majority of genes showing negative regulation in bees from HIC. In particular, the marked transcriptional down-regulation of a member of the NF-κB gene family indicates that the pathogen-parasite interaction can interfere with a number of immune responses regulated by this transcription factor, such as the synthesis of antimicrobial peptides, clotting, melanisation and antiviral defences [38]–[45]. Furthermore, the marked impact on some serine proteases seems to reinforce the virus-mite effect on humoral components of the immune response. Even though we have limited information on the role of these enzymes in honeybee immunity [38], [51], we can reasonably assume, on the basis of studies on other model insects, that the down-regulation of these genes may well impair the enzymatic cascades leading to the activation of melanisation and clotting responses [52], [53], as well as other immune pathways that remains to be further characterized.

This immunosuppressive effect seems to be largely driven by viral replication, since we have observed that *Varroa* feeding alone does not seem to influence the expression level of *dorsal-1A*, the most down-regulated gene that we used as an indicator of immunosuppression, in our *in vitro* infestations with mites on bee larvae, either bearing or not a DWV infection. This finding is further corroborated by a recent genome-wide analysis of the transcriptional profile in bees infested by the *Varroa* mite, but not infected by viruses, which evidenced a clear differential expression, with respect to control uninfested bees, only for genes involved in metabolic processes and nerve signalling [54]. Collectively, these experimental evidences indicate that DWV may use a conditional virulence strategy which disrupts NF-κB immune signalling. However, a more direct experimental approach is needed to assess the impact of DWV infection on the bee immunosuppression syndrome and to follow its dynamic changes with the progression of viral infection.

The significant increase of DWV genome copies in response to *dorsal-1A* knockout by RNAi shows that this gene plays a crucial role in the antiviral immune response controlling DWV replication, and corroborates the hypothesis that DWV adopts a conditional virulence strategy partly based on the transcriptional down-regulation of this NF-κB family member. Many viruses target this key-molecule, which is central in the orchestration of the complex network of responses to infection and, more generally, to environmental stress [55]–[57]. However, the present case seems to be different, as, unlike other viruses infecting vertebrates [56] or invertebrates [57], DWV would exert a transcriptional down-regulation, which results in a reduced level of NF-κB transcripts. This suggests that any bee antiviral immune response relying on this transcription factor is reduced, but not strongly suppressed, as happens in more aggressive viral pathogens, which are able to interfere with NF-κB, either directly or indirectly, by targeting upstream events that control NF-κB activation [56]–[59]. Therefore, the delicate balance of covert DWV infections could be disrupted by any stress factor that activates a response triggered by NF-κB. In other words, the limited availability of this transcription factor seems to be sufficient to maintain under control the DWV infection, which, however, may undergo intense replication if NF-κB is substantially subtracted by any other pathway activated by acute responses to stress factors.

In insects, wounding activates NF-κB dependent clotting and melanisation [39]; therefore, the bee reaction to *Varroa* feeding wounds is expected to use the already limited cellular pool of this transcription factor in DWV infected individuals, and consequently can promote an intense viral replication, which can be further aggravated by the injection of additional virus particles.

In this framework, the observed viral replication triggered by injection of bacteria in DWV infected bees, rather than to be exclusively considered a consequence of a wide antimicrobial immunosuppression induced by mite feeding [24], could be partly reinterpreted as a possible effect of the competitive use of this transcription factor involved in multiple immune responses [38]–[45], and available at reduced level in infected bees. Indeed, we have observed that the immunosuppression syndrome is unexpectedly characterized by the up-regulation of a limited number of genes (Eater-like, NimC2, serine proteases), that are mostly involved in bacterial phagocytosis [60]. The up-regulation of Eater-like has also been reported in bees infected by IAPV [61]. This experimental evidence suggests that a complete suppression of the bee antimicrobial response is not a stringent functional requirement of the complex co-evolutionary process among bees, DWV and *V. destructor*.

The DWV-mediated immunosuppression of NF-κB signalling may provide significant benefits to the vector mite, because it reinforces the disruption of immune reactions activated by feeding wounds and salivary components [62]. This could be particularly relevant to the *Varroa* mite since both the invading mite and its offspring feed through the same hole made in the honeybee cuticle, at the beginning of the pupal stage, by the mother mite [63]: clotting and melanization could severely impair mite feeding activity. We reasonably speculate that the *Varroa*-DWV association can be interpreted as a mutualistic symbiosis in its early stages. A similar, but more ancient, evolutionary pattern can be observed in some parasitoids of lepidopteran larvae, which are associated with immunosuppressive viral symbionts in the family Polydnaviridae [64]. The ancestor of bracoviruses, members of the polydnavirus family, is a host pathogen of the Nudivirus group, closely related to baculoviruses, which was domesticated by the wasp to its own benefit [65]. The “alliance” of parasitic organisms with the viral pathogens of the host seems to be an effective strategy also for some insects attacking plants. The tight association between stylet feeding insects and viral plant pathogens provides a good example of how these latter can be used for suppressing the plant defense response against them. Indeed, it has been recently demonstrated that the Cucumber mosaic virus (CMV) encodes a protein that disrupts the plant antiviral mechanisms, and, at the same time, blocks defense pathways active against aphids [66]. These are just a few examples of the multifaceted viral mutualistic symbioses, which have played an important role in life evolution, by allowing a more effective exploitation of hostile ecological niches [67].

In order to investigate the dynamical properties of our system, we built and analysed a series of dynamical models capturing differing assumptions on the interactions between virus, host and additional stressors (e.g. mite infestation), and contrasted the model behavior with our observed results. This methodology allowed us to conclude in favour of a threshold immune-suppression model for DWV, which would allow the virus to function as an opportunistic pathogen, able to switch in response to host condition from a stable, cryptic state to aggressive exploitation. This opportunistic strategy is highly reminiscent of the condition-dependent behaviour of temperate phage viruses, which are able to switch between cryptic vertical transmission and aggressive horizontal transmission, as a function of the stress level (SOS response) in their bacterial host [68]. Clearly, the mechanisms underlying condition-dependent host exploitation are vastly different between phage lambda and DWV, however the selective contexts contain analogies: aggressive exploitation and increased horizontal transmission is likely to be more favourable when current host condition dips below a critical limit - broader biological examples of rats leaving a sinking ship. This novel dynamical framework builds on our experimental results and offers predictions for future work. Specifically, not only *V. destructor* but other stressors competing for immune resources have the potential to destabilise DWV dynamics by tipping DWV copy number above its control threshold into its aggressive exploitation regime.

**Figure 7:**
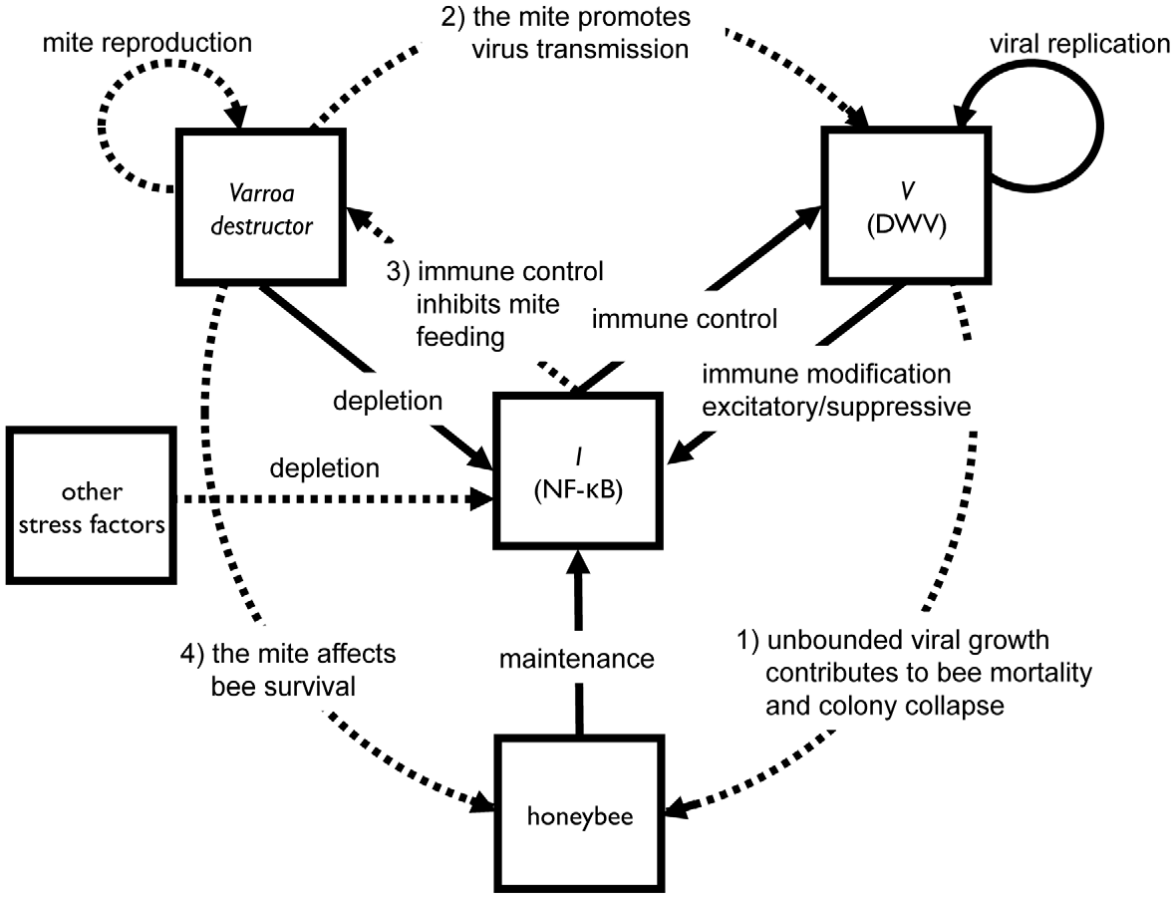
Schematic diagram of within-host viral copy number (*V*) and immune currency (*I*) dynamics. The bold lines represent dynamical processes captured explicitly in equations S4, S5. In this model, the viral population dynamics are governed by two antagonistic processes, replication and control (by the immune system). The immune dynamics are in turn governed by three processes; maintenance (increasing immune stocks), stressors (depleting immune stocks) and a specific impact of virally-mediated immune modification (ranging from excitatory to suppressive). The dotted lines represent processes that are external to the model: 1) over-growth of the virus directly leads to increased bee mortality and collapse of the colony (Figures 1 and 2); 2) despite impending collapse within a focal colony, the virus can escape its host via horizontal transmission facilitated by its mite symbiont [21], [73]; 3) the mite may gain further advantages from its association with an immuno-suppressive virus, as the suppression will further release immunological control of mite feeding; 4) the mite can affect honeybee survival [21].

The key immune currency identified by our transcriptome analysis is a member of NF-κB gene family. This gene family not only plays a central role in insect immunity [69], but is also involved in intricate cross-talks with a number of physiological and stress response pathways, conserved across different organisms [55], which are often reciprocally tuned to allow optimal energy allocation between metabolism and immune response, as recently demonstrated in *Drosophila* [70]; the observed induction of DWV replication in bees exposed to cold stress [50] seems to lend further support to this hypothesis. Therefore, different stress factors impacting immunity and metabolism may compete for the use of NF-κB cellular pools, already reduced by the parasite-pathogen association, promoting intense viral replication in bees harbouring silent infections and subsequent colony collapse (Figure 7). The considerable diversity of stress factors that can interfere with the immune system may partly account for the variety of putative causal agents invoked so far to explain honeybee colony losses, that do not seem to be univocally linked to a specific causative agent.

## Materials and Methods

### Population Sampling

Two apiaries, made of six colonies each, were set up at the end of April in an isolated location of the Prealps (Porzus, Udine, Italy; 46°11′N, 13°20′E), 1.6 km apart from each other. Previous studies indicated that the local bee population consists of hybrids between *A. mellifera ligustica* and *A.m. carnica* [71], [72]. Queens were local and naturally inseminated; hives were all treated the preceding year with acaricides, in order to have very low starting populations of the parasitic mite *V. destructor* at the beginning of the experiment.

In one apiary mite populations were kept under control during the experiment by treating the hives with prophylactic acaricides (the colonies of this apiary are referred to as “low infested colonies” (LIC) in the text). A thymol-based product in tablets (ApiLife Var) was used, from mid-August to mid-September, in presence of brood; at the end of October, two treatments were carried out with oxalic acid, in absence of brood (5 ml of a solution of 30 g of oxalic acid in 1 l of deionized water were sprayed on both sides of each comb of the hive). In the other apiary no acaricidal treatments were carried out (the colonies of this apiary are referred to as “highly infested colonies” (HIC) in the text). In August, one hive in this apiary succumbed because the queen became drone layer, and was not further considered.

Re-infestation can strongly affect the population dynamics of the mite if highly infested, weak colonies are robbed by low infested, strong colonies located in the vicinities [73]. Therefore, we adopted an experimental design in which treatments (high and low infestation) were applied to hives belonging to two different apiaries located at a distance such that the environmental conditions were the same but robbing was prevented. As regards as possible factors affecting the independency of hives, with similar infestation rates, belonging to the same apiary (e.g. worker drifiting), available data suggest that they should not affect significantly any of the variables considered in the field study [74].

This experimental design was conceived to allow a very detailed and direct analysis of the potential collapse-inducing factors, under uniform experimental conditions. Severe logistical constraints precluded the use of multiple apiaries per treatment in the field experiment; however, the laboratory experiments had a replicated design and confirmed the central field observations.

Bee population in the experimental hives was estimated approximately once a month, from May to October, by counting the number of full or partial “sixth of frames” covered by bees in each hive at sunset and calculating the overall bee population, on the basis of the correlation which indicates that one fully covered sixth of comb corresponds to 253 adult bees [75]. The number of brood cells was estimated using the same method, taking into account that one sixth of frame of brood cells corresponds to 728 worker brood cells.

On the same sampling dates, starting in June, the infestation of adult bees and brood by *Varroa* mites was estimated. The first was assessed on a sample of about 1,000 bees, collected from a frame located in the central part of the hive. Sampled bees were transferred into a flask, covered with 35∶65 ethanol∶water, and hand-shaken for about 5 minutes. Then, bees were recovered by filtration, the liquid phase was filtered again with a convenient sieve to collect the mites and reused for rinsing the bees until no mites were found in three consecutive washes. Infestation level was expressed as number of mites per adult bee.

To assess brood infestation, one piece of brood comb (10×10 cm) was collected from each colony and 50 sealed cells from each side were opened and inspected for the presence of mites. Only dark-red mites were considered, to exclude any offspring produced by founder mites. Infestation was expressed as number of mites per cell.

Mite infestation was calculated using the following formula: [(adult bees infestation×bee population)+(brood infestation×brood cells)]×1,000/(bee population+brood cells).

Dead bees found in cages placed in front of the colonies were counted on weekly basis, from May to October. Bee mortality on each sampling occasion was calculated by averaging the number of dead bees in the time interval elapsed since the last sampling date; this value was then referred to the mean bee population in that period, obtained by considering the initial and final bee population and then multiplied by 1,000.

### Metagenomic Survey of Microrganisms in the Hives

Samples of 10 bees were collected in October, from each LIC and HIC colony (n = 6 and n = 5 for the two groups respectively), ground in liquid nitrogen and immediately used to extract total RNA, using Tri-Reagent (Ambion Inc.). These RNA samples were processed using the TruSeq mRNA-seq sample prep kit (Illumina, Inc., CA, USA) starting from 2 micrograms of total RNA. Briefly, poly-A containing mRNA molecules were isolated using poly-T oligo-attached magnetic beads using two rounds of purification. During the second elution of the poly-A RNA, the RNA is also fragmented and primed for cDNA synthesis. Then standard blunt-ending plus add ‘A’ was performed and Illumina adapters with indexes (from 1 to 12) were ligated to the ends of the cDNA fragments. After ligation reaction, separation of not ligated adapters and size selection in the range 500–600 bp was performed on 2% low-range agarose gel. Samples were amplified by PCR to selectively enrich those DNA fragments in the library having adapter molecules at both ends.

Pools of 3–4 samples were loaded on cBot, to create clusters and sequenced at ultra-high throughput [76] on HiSeq2000 (Illumina Inc.). One lane for 12 samples was run obtaining 23–36 millions of pair-end reads per colony, 100 bp long.

Sequences from each colony were quality trimmed by CLC (modified-Mott trimming algorithm, trim using quality score 0.03) and mapped on Amel 4.0 bee genome reference sequence using CLC Genomics Workbench (CLCBio, Denmark). The un-aligned reads (about 20% of total reads) were de novo assembled using the same software. Contigs were compared to the non-redundant sequence databases at NCBI (http://www.ncbi.nlm.nih.gov), using BLASTX (protein homology). BLASTX alignment hits with e-values larger than 1×10^−5^, scores lower than 100 and percentage identity lower than 50% were filtered; isolated assignments (i.e. taxa hit by one sequence only) were discarded. Custom applications written in Perl were used to parse the results. Raw pair-end sequences used for metagenome survey are available at: https://services.appliedgenomics.org/sequences-export/193-Nazzi_et_al/; password: rawdata).

To get a description of the microorganisms associated to the bees under study, taking into account all taxa whose presence was not just sporadic, we considered only those represented in at least two colonies of either group of hives. The resulting list was then filtered against available data on honeybee symbionts from previous reports [11], [35], [77]–[79] retaining only taxa whose presence in honeybees had already been demonstrated.

### PCR Analysis of Bee Viruses

Total RNA was isolated from individual bees by using Trizol Reagent (Invitrogen, Carlsbad, CA), according to the manufacturers' instructions. The concentration and the purity of total RNA was determined using a spectrophotometer (Nanodrop ND100, Thermo Scientific Inc.).

Virus presence was assessed by conventional RT-PCR as described elsewhere [80] using the primer pairs reported in Table S3.

The quantification of DWV genome copies in individual bees was performed by SYBR-Green Real-Time Quantitative RT-PCR. The amplification conditions and reaction mixture were the same as conventional RT-PCR, using QuantiFast SYBR Green RT-PCR Kit (Quiagen, Hilden, Germany). The titers of DWV were determined by relating the *C*_T_ values of unknown samples to an established standard curve, according to the absolute quantification method previously reported [81]. The standard curve was established by plotting the logarithm of seven 10-fold dilutions of a starting solution containing 21.9 ng of plasmid DNA (TOPO TA Cloning) with DWV insert (from 21.9 ng to 21.9 fg), against the corresponding threshold value (*C*_T_) as the average of three repetitions. The PCR efficiency (E = 107.5%) was calculated based on the slope and coefficient of correlation (R^2^) of the standard curve, according to the following formula: E = 10^(−1/slope)^−1 (Slope = −3.155, Y-intercept = 41.84, R^2^ = 0.999).

### Effect of *Varroa* Mite feeding on Viral Replication in Honeybee Larvae

This experiment was designed to assess the impact of *Varroa* mite feeding on DWV replication in honeybees.

Bees and mites used in this and the following laboratory experiments came from *A. mellifera* colonies maintained in Udine (northeastern Italy). Previous studies indicated that the local bee population consists of hybrids between *A.m. ligustica* and *A.m. carnica* [71], [72].

The mites and last instar bee larvae were collected from brood cells capped in the preceding 15 h obtained as follows. In the evening of the day preceding the experiment the capped brood cells of a comb were marked. The following morning the comb was transferred to the lab and unmarked cells, that had been capped overnight, were manually unsealed. The comb was then placed in an incubator at 35°C, 75% R.H. where larvae, either infested or not, spontaneously emerged.

Last instar bee larvae were transferred into gelatin capsules (Agar Scientific ltd., 6.5 mm diameter) with 1 or 3 mites, and maintained at 35°C, 75% R.H. for 12 days [82]; *Varroa*-free larvae were used as controls (Figure S4). After 1, 6 and 12 days, 5 bees for each infestation level were sampled to determine the total number of DWV genome copies, as described above.

### Effect of Artificial Virus Infection on the Survival of Adult Bees

This experiment was designed to assess the longevity of adult bees emerging from larvae that received an injection of different numbers of DWV genome copies.

The artificial infection with DWV of last instar bee larvae, collected as described above, was performed by injecting 2 µl of a lysate of symptomatic bees, at two different dilutions, using a Hamilton syringe equipped with a 30 gauge needle. Five bees with crippled wings, that is the typical symptom of DWV infection, collected in mite-infested colonies, were frozen in liquid nitrogen, crushed with a pestle in a mortar and suspended in 5 ml of phosphate buffer solution, pH 7.4. After centrifugation (3,000 rpm/min for 30 min at 4°C), the supernatant was transferred into sealed tubes and stored at −20°C until use [83]. The extract of five healthy bees was prepared in the same way and used for control injections.

The lysates obtained as above were tested for seven honeybee viruses and two fungi species by conventional RT-PCR as described elsewhere [80] using the primer pairs reported in Table S3.

The number of DWV genomic copies in the samples was assessed by Real-Time Quantitative PCR, as described above. The two adopted dilutions (10^−3^ and 10^−5^) in PBS allowed the delivery of an estimated number of DWV genome copies of 1.66×10^3^ and 16.6, respectively.

Following injection, bee larvae were confined into gelatin capsules, as described above, and maintained in an incubator at 35°C, 75% R.H.. After 12 days, when adult bees were fully developed, gelatin capsules were opened and the experimental bees transferred to an aerated plastic cage (18.5×10.5×8.5 cm), maintained in an incubator, at the same condition indicated above, and fed *ad libitum* with sugar candy (Apifonda) and water. The number of bees with deformed wings was recorded and dead bees were daily counted and removed. The experiment was replicated 3 times, across April–May, using 25–30 bees per replicate of each treatment. The proportion of symptomatic bees among those treated as above confirmed the effective infection by this method (Figure S5).

### RNA-seq

The same RNAs used for metagenomic analysis were analyzed in terms of gene expression. The standard mRNA sample prep from Illumina was used to produce 36 bp long tags, about 25–30 millions per sample. CLC-Bio Genomics Workbench software (CLC Bio, Denmark) was used to calculate gene expression levels based on Mortazavi et al. approach [84]. A table reporting the data used for subsequent analysis can be found at: https://services.appliedgenomics.org/sequences-export/193-Nazzi_et_al/ (username: nazzi_et_al; password: rawdata).

Differential expression of six selected genes was confirmed by means of Quantitative Real-Time RT-PCR using the primer pairs reported in Table S3. Relative gene expression data were analyzed using the 2^−ΔΔ*C*_T_^ method [85]. To assess that the amplification efficiencies of the target and reference gene (β-actin) were approximately equal, the amplification of six five-fold dilution of total RNA sample (from 1,000 ng to 0.32 ng per reaction) were analysed; in all cases the efficiency plot for log input RNA versus Δ*C*_T_ had a slope lower than 0.1 (Dorsal = 0.089; cSP33 = 0.019; SPH51 = 0.025; Eater-like = 0.064; NimC2 = 0.035; PGRP-S2 = 0.048). The calibrator was the LIC group. Three estimates of the ΔΔ*C*_T_ of each gene were obtained from independent analyses; for each analysis, one pool of three bees from each colony of both groups was used.

The differential expression of *dorsal-1A*, the most down-regulated gene with potential impact on several immune and stress responses, was also confirmed by absolute quantification; in this case, one pool of three bees from five colonies of both groups was analysed.

The standard curve was established by plotting the logarithm of nine 10-fold dilutions of a starting solution containing 127.4 ng of plasmid DNA (TOPO TA Cloning) with *dorsal-1A* insert (from 127.4 ng to 1.3 fg), against the corresponding threshold value (*C*_T_) as the average of three repetitions. The PCR efficiency (E = 93.2%) was calculated based on the slope and coefficient of correlation (R^2^) of the standard curve, according to the following formula: E = 10^(−1/slope)^−1 (Slope = −3.495, Y-intercept = 46.19, R^2^ = 0.996).

### Effect of *Varroa* Infestation and Virus Infection on the Expression of *dorsal-1A*

In order to assess the role of the *Varroa* mite and DWV in the transcriptional down-regulation of *dorsal-1A*, we measured the impact of mite feeding on the expression level of this gene in virus-free bee pupae and pupae testing positive for DWV. Honeybee pupae, either uninfested or infested by one mite, were prepared as described above, then, after 12 days, they were processed for Quantitative Real-Time RT-PCR, to evaluate the expression of *dorsal-1A* and DWV infection rate in infected bees. To increase the chances of sampling DWV-free bees, the experiment was carried out on three dates in early Spring, when, according to the data shown in Figure 2B, the prevalence of infection is low, and repeated twice later in the year, when most bees test positive for DWV. Thus virus free and virus infected bees had to be collected on different times; however, a regression analysis revealed no significant effect of time on Dorsal expression in virus free bees.

### RNAi

Double-stranded honeybee *dorsal-1A* (*A. mellifera* Dorsal variant A, mRNA, GI:58585243, 2389 bp) was prepared using MEGAscript RNAi kit (Ambion), following the manufacturer's standard protocol. The target sequence was PCR amplified with specific primers, carrying a 5′ tail of the T7 promoter at both ends and used as template for T7-*depended in-vitro* transcription. Primers used were:

F-5′-TAATACGACTCACTATAGGGAGACAATCCAGCACTTATTC-3′;

R-5′-TAATACGACTCACTATAGGGAGCCTGAATAGTGTTATTAGC-3′.

The reaction product was subjected to DNase digestion, purified and the final preparation was dissolved in nuclease free-water.

Individual frames were removed from the colony and stored in an incubator overnight, at 34°C, 90% R.H.. Emerging bees were maintained as groups of 30 individuals in sterile boxes, as described by Evans et al. [86]. Experimental bees were fed daily with 2 ml of a 50% sucrose/protein solution, containing 50 µg of dsRNA of *dorsal-1A*, while controls were fed with a similar solution, containing a dsRNA of mGFP6 (Green Fluorescent Protein), obtained as described above. Samples of 5 bees were collected at the beginning of the experiment, to assess the starting level of scored parameters, and after 48 and 96 hours of exposure to the dsRNA feeding solution. Samples were stored at a −80°C, until use for RNA extraction.

The transcription level of *dorsal-1A* and the number of DWV genome copies were determined by SYBR-Green Real-Time Quantitative RT-PCR, as described above.

### Statistical Analysis

Comparisons between treated and untreated colonies, for bee population, bee mortality, *Varroa* mite infestation and gene expression values resulting from RNA-seq, were carried out using the non-parametric Mann-Whitney test. In all cases, the number of replicates in each group correspond to the number of colonies, that was 6 for the low infested group (LIC) and 5 for the highly infested one (HIC).

To compare both the mortality rates and the infestations in the two groups of colonies while controlling for the correlation among repeated observations on the same colony over time, a model for longitudinal data was estimated; in this case a total of 44 observations, deriving from 11 colonies, observed 4 times each, were considered. A between groups regression panel model pointed out a significant effect of the indicator variables (bee mortality and mite infestation respectively) for HIC (bee mortality: estimated coefficient 3.045, *P* = 0.005; mites/1,000 bees: estimated coefficient 116.968, *P* = 0.005).

The proportion of DWV infected bees, out of the total analyzed in LIC and HIC, was compared using the Fisher Exact Solution test. In this case, 5 bees per group and per date were used for the analysis.

The number of DWV genome copies in individual honeybees, from LIC and HIC, was compared with the non-parametric Mann-Whitney test. In this case, 6 and 13 bees from LIC and HIC, respectively, were used in September, while, in October, 14 and 11 bees were considered for the same experimental categories.

Data from the experiment on the effect of *Varroa* mite feeding on viral replication in honeybee larvae were analyzed using the Scheirer-Ray-Hare extension of the Kruskal-Wallis test. Data from 5 bees per infestation level per time after the beginning of the experiment were used. Comparison of survival rates following injection of bee body lysates were carried out using the logrank test without continuity correction; in this case, 25–30 bees per group were used in each of the 3 replicates.

Data on gene expression in virus free and virus infected bees either infested or not by the *Varroa* mite were compared with the GLM procedure after log tranforming data; 9 uninfested and 9 infested virus-free bees, 10 uninfested and 10 infested virus infected bees were used for the analysis; the software Minitab was used.

In RNAi experiments, gene expression and viral replication in bees fed with dsRNA of *dorsal-1A* or dsRNA of Green Fluorescent Protein, as a control, were compared using the Scheirer-Ray-Hare extension of the Kruskal-Wallis test. Five bees per each time per treatment were used in the analysis.

### Theoretical Analysis

A characteristic of DWV infections in unstressed hosts is the ability of the virus to persist in a cryptic state, and to be stably transmitted vertically [29]. We use the existence of a stable state of chronic infection to base our dynamical model on a ‘predator-prey like system’ [46], as described by the following equations for viral copy number (*V*) and shared immune currency (*I*),
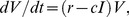
(S1)

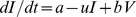
(S2)


These equations (identical to equations 1 and 3 in [46]) describe the within-host growth of a pathogen population *V* and its controlling immunological counterpart *I*. The maximal rate of pathogen replication is *r*, which is countervailed by a rate of immunological control *cI*. The dynamics of *I* are shaped by an intrinsic production rate *a*, a rate of decay *u* and an activation rate *bV* (activation by the pathogen population). A stability analysis of equations S1, S2 using standard techniques [87] and assuming all parameters are positive, reveals that whenever pathogens are able to invade a naïve host (when *r*>*ca/u*) then their density *V* will tend to a single stable equilibrium at 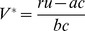.

A key characteristic of the interaction between DWV and its host is some degree of immuno-suppression (Table 1). The simplest modification of equation S2 to allow for immuno-suppression is to consider the negative space of the ‘immune-activation’ parameter *b*. If *b* is negative, then increasing pathogen density *V* will act to reduce the immunological control variable *I*, with potentially de-stabilising consequences. Accordingly, a stability analysis now reveals that whenever pathogens can invade a host (same condition as above), their density will always increase without bounds, thus we have an obligately virulent pathogen that will grow and consume any host that they are able to establish within.

We now turn to our threshold suppression model. We again assume that the dynamics of *I* are modified by an interaction with the pathogen population *V*, however we now assume that the sign of the interaction (immuno-stimulatory or immuno-suppressive) will depend on the magnitude of *V*. Specifically, we assume that at low densities the pathogen is a net activator of immunological activity, whereas at high densities (whenever *V*>*b/s*) the pathogen becomes immuno-suppressive, with *b/s* controlling the threshold point between the two regimes. These assumptions give the following revised equation for the dynamics of *I*

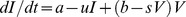
(S3)


To clarify presentation, we first normalize the system (S1,S3) to reduce the parameter dimensions. Specifically, we rescale the units of time to the maximal growth rate of the virus (*t′ = rt*), the units of viral density to the density that halts immune proliferation (*V*′ = *Vs/b*) and the units of immune density to the density that halts viral proliferation (*I′ = Ic/r*). Applying these normalizations to equations (S1,S3) lead to the following equations
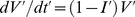
(S4)

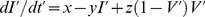
(S5)


Note that the full system (S1,S3) can be recovered from (S4,S5) by rescaling the units and replacing parameters as follows: 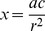, 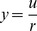, 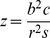. A stability analysis of the system (S4,S5) reveals equivalent invasion conditions (*1>x/y*) but following invasion the virus can either tend to a stable equilibrium at 
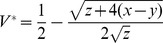
 (solid line in Figure 6), or grow without limit if *V* is above an unstable equilibrium at 
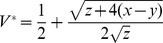
 (dashed line in Figure 6) or if non-viral immunological depletion is sufficiently high (if y>x+z/4, to right of intersection in Figure 6). For low *y* (*y<x+z/4*), the stable equilibrium 
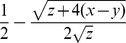
 is an increasing function of *y* and the unstable equilibrium 
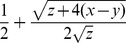
 is a decreasing function of *y*.

## Supporting Information

Figure S1 **Proportion of bees infected by different viruses in highly and low infested colonies.** BQCV: Black queen cell virus, SBV: Sacbrood virus. Error bars indicate the standard deviation. Both BQCV and SBV prevalence fluctuated and overall declined over time.
(TIF)

Figure S2 **Relative gene expression of six immunity genes, by Quantitative Real-Time RT-PCR, in honeybees from highly infested colonies.** The 2^−ΔΔ*C*_T_^ of each gene ± the standard deviation is reported; the horizontal line represents the reference level in low infested colonies: gene expression values below the line are down-regulated, values above the line denote up-regulation. Real-Time RT-PCR data confirmed the differential expression of selected immunity genes detected late in the season by RNAseq analysis in bees from highly infested colonies.
(TIF)

Figure S3 **Dorsal expression in low and highly infested colonies.** Dorsal copies in honeybees, collected in October from low and highly infested colonies. The error bars indicate the standard deviation; the reported difference is statistically significant (Mann Whitney test: U = 2, n_1_ = n_2_ = 5: *P*≤0.05). Dorsal expression was reduced in bees from highly infested colonies in October.
(TIF)

Figure S4 **Honeybees obtained from larvae infested or not by the parasitic mite** ***V. destructor*** **and maintained in gelatin capsules until the completion of their development.** In the capsule on the right, a mite can be noted on an infested bee which shows a short abdomen, induced by the viral infection.
(TIF)

Figure S5 **Proportion of bees showing the characteristic symptom of DWV after artificial infection at the larval stage.** Bee larvae received an injection of two different dilutions (1∶1,000 and 1∶100,000) of a whole body lysate obtained from bees with deformed wings (DW), and a diluted lysate (1∶1,000) of bees with normal wings (NW) as a control. The error bars indicate the standard error. The dose-response relationship between the injected dose and the proportion of symptomatic bees confirms the efficiency of the infection method.
(TIF)

Table S1 **Closest sequenced relatives identified, through BLAST analysis of the high-throughput sequence data, in the colonies under study, in October.** Only taxa that were present in at least two colonies of either group of hives are reported. The number of hits and the average percentage identity are reported for each taxon.
(PDF)

Table S2 **Relative expression of honeybee immune genes in highly infested colonies.** For each highly infested colony (HIC1 to HIC5), the RPKM ratio between that colony and the average of low infested colonies is reported; red, pink, yellow and green are used to denote genes whose ratio was lower than 0.5, between 0.5 and 0.9, between 1.1 and 2, higher than 2, respectively. Gene list from [38]; only genes with no-zero reads in at least one colony of each group are reported. Most genes were down-regulated in highly infested colonies, the most marked effect was noted for members of the Toll pathway and Serine Proteases.
(PDF)

Table S3 **Primer pairs used for conventional RT-PCR or Quantitative Real-Time RT-PCR analyses.**
(PDF)
