# Supplementary figures and images for: Synergistic Parasite-Pathogen Interactions Mediated by Host Immunity Can Drive the Collapse of Honeybee Colonies
Source: PLoS Pathog. 2012 Jun 14;8(6):e1002735. doi: 10.1371/journal.ppat.1002735 (PMC3375299; doi:10.1371/journal.ppat.1002735)

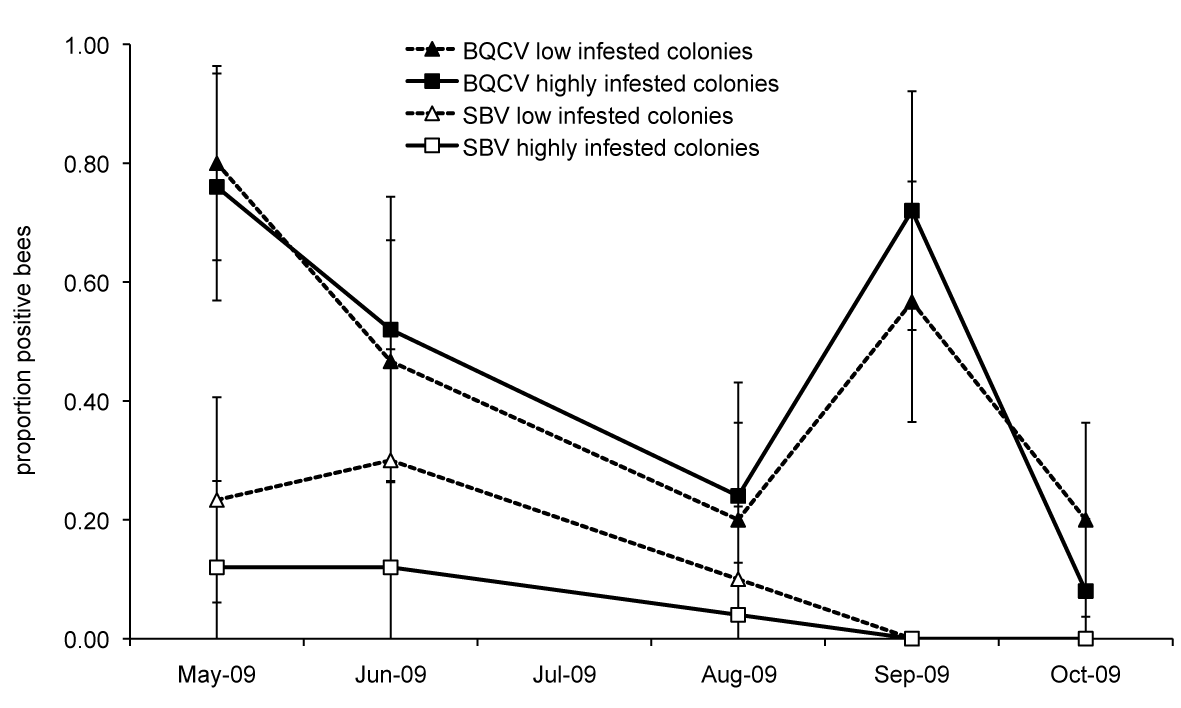

Supplement: Figure S1 — Proportion of bees infected by different viruses in highly and low infested colonies. BQCV: Black queen cell virus, SBV: Sacbrood virus. Error bars indicate the standard deviation. Both BQCV and SBV prevalence fluctuated and overall declined over time. (TIF) [file ppat.1002735.s001.tif]

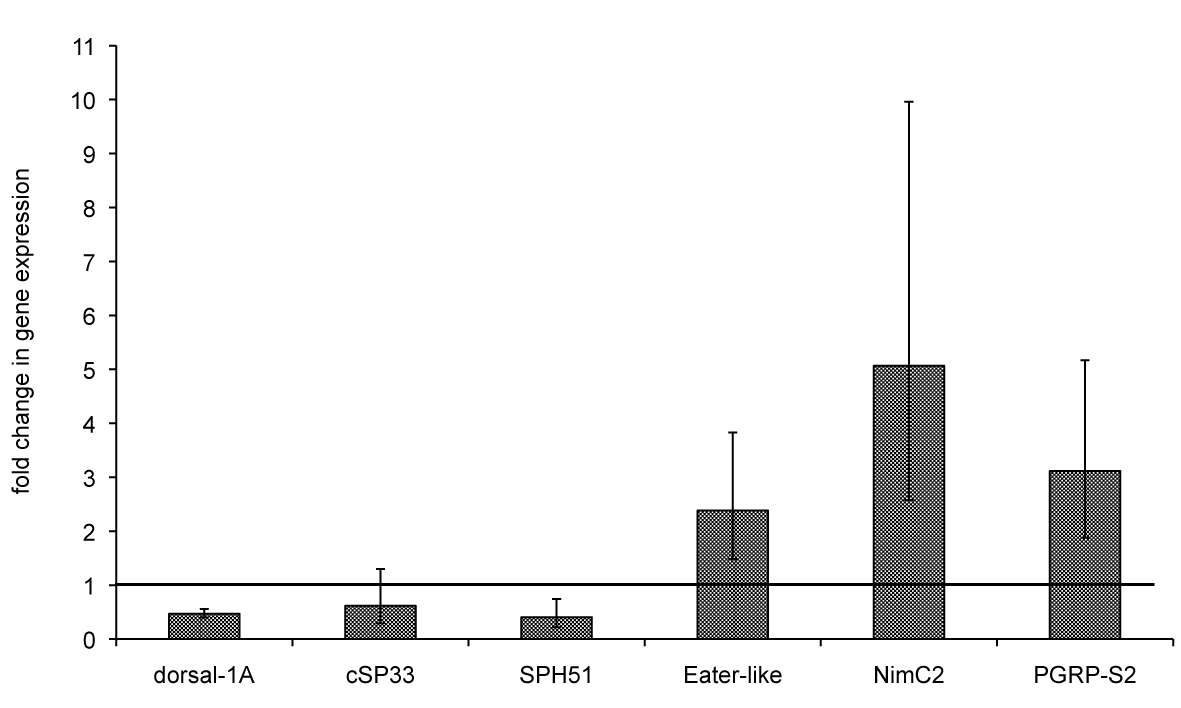

Supplement: Figure S2 — Relative gene expression of six immunity genes, by Quantitative Real-Time RT-PCR, in honeybees from highly infested colonies. The 2−ΔΔCT of each gene ± the standard deviation is reported; the horizontal line represents the reference level in low infested colonies: gene expression values below the line are down-regulated, values above the line denote up-regulation. Real-Time RT-PCR data confirmed the differential expression of selected immunity genes detected late in the season by RNAseq analysis in bees from highly infested colonies. (TIF) [file ppat.1002735.s002.tif]

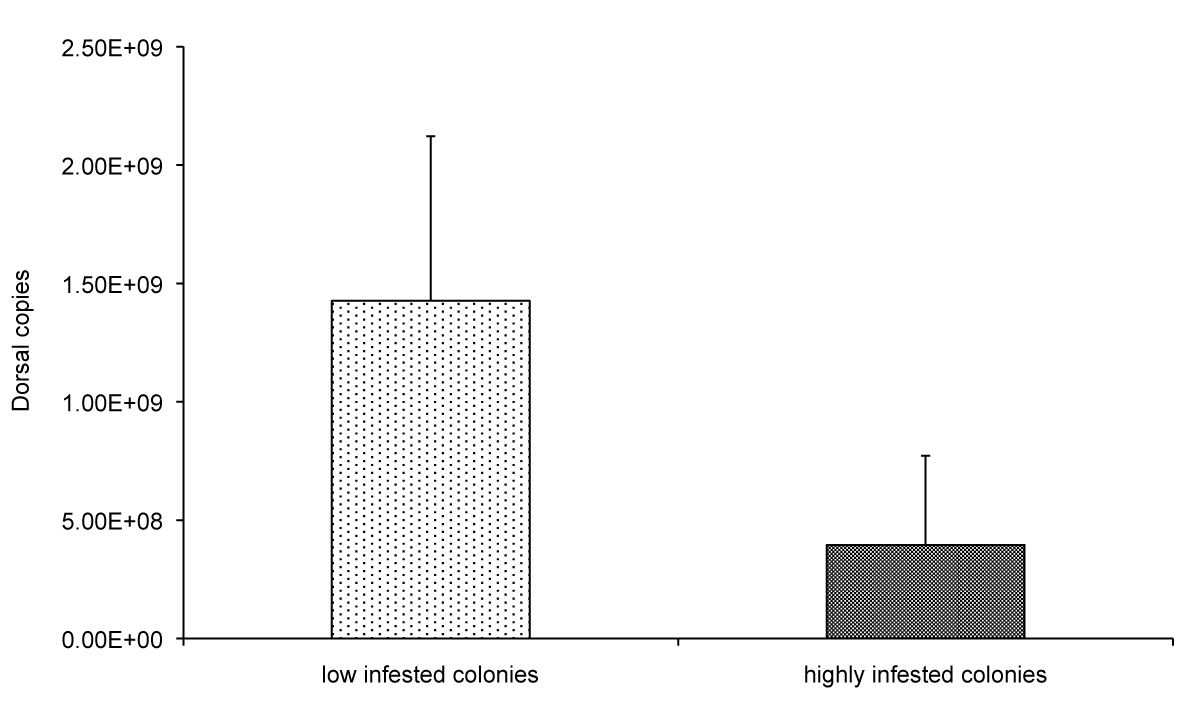

Supplement: Figure S3 — Dorsal expression in low and highly infested colonies. Dorsal copies in honeybees, collected in October from low and highly infested colonies. The error bars indicate the standard deviation; the reported difference is statistically significant (Mann Whitney test: U = 2, n1 = n2 = 5: P≤0.05). Dorsal expression was reduced in bees from highly infested colonies in October. (TIF) [file ppat.1002735.s003.tif]

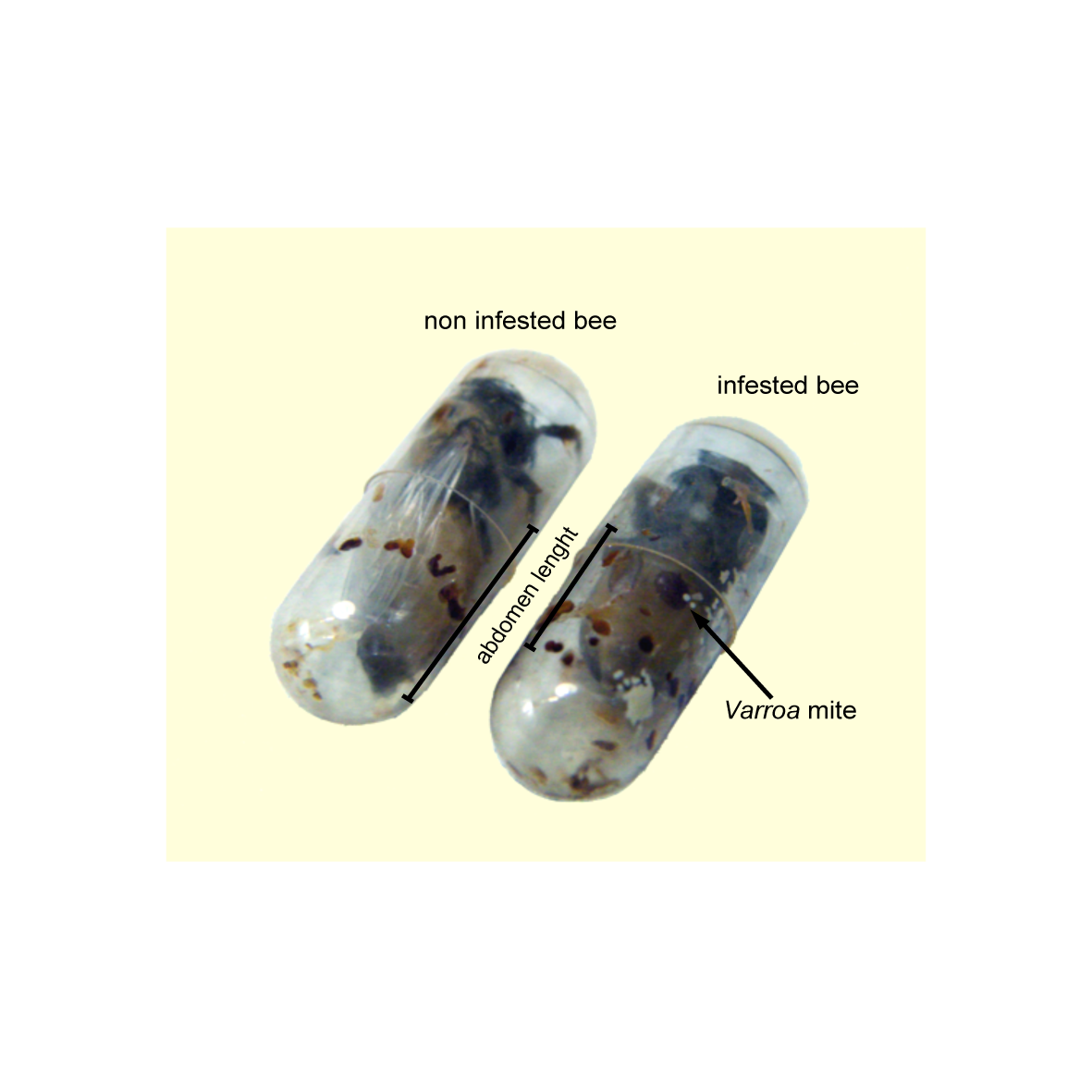

Supplement: Figure S4 — Honeybees obtained from larvae infested or not by the parasitic mite V. destructor and maintained in gelatin capsules until the completion of their development. In the capsule on the right, a mite can be noted on an infested bee which shows a short abdomen, induced by the viral infection. (TIF) [file ppat.1002735.s004.tif]

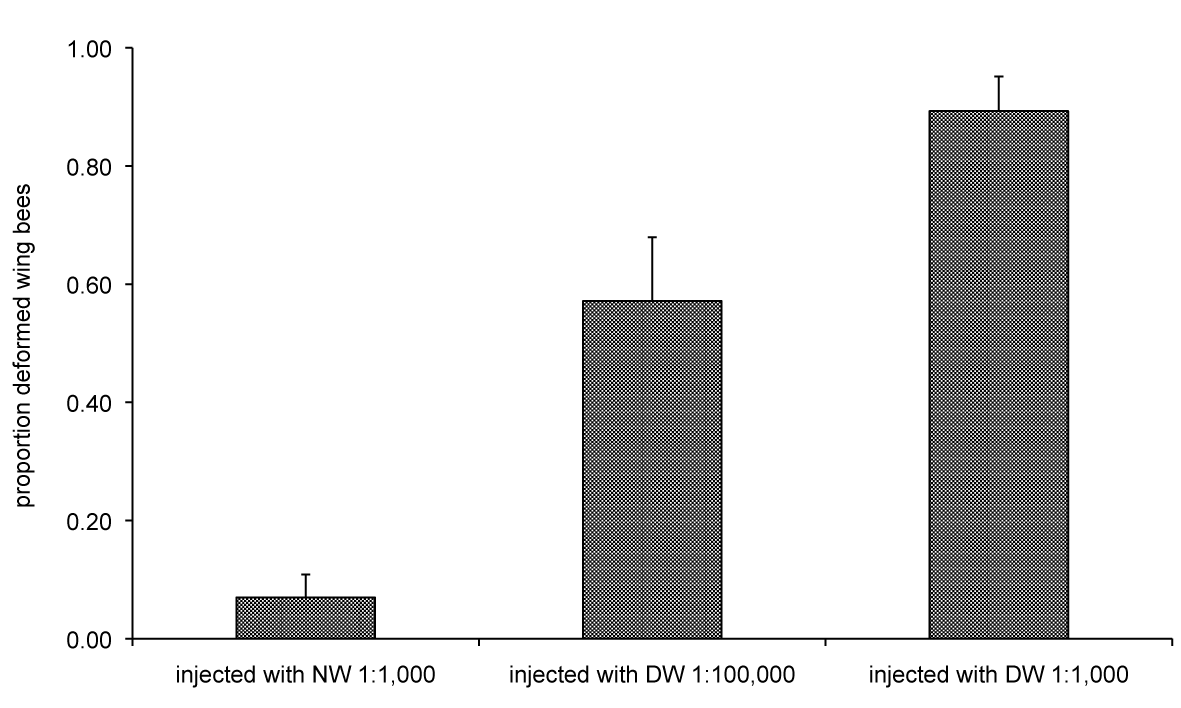

Supplement: Figure S5 — Proportion of bees showing the characteristic symptom of DWV after artificial infection at the larval stage. Bee larvae received an injection of two different dilutions (1∶1,000 and 1∶100,000) of a whole body lysate obtained from bees with deformed wings (DW), and a diluted lysate (1∶1,000) of bees with normal wings (NW) as a control. The error bars indicate the standard error. The dose-response relationship between the injected dose and the proportion of symptomatic bees confirms the efficiency of the infection method. (TIF) [file ppat.1002735.s005.tif]
