# Supplementary material for: Synergistic Parasite-Pathogen Interactions Mediated by Host Immunity Can Drive the Collapse of Honeybee Colonies
Source: PLoS Pathog. 2012 Jun 14;8(6):e1002735. doi: 10.1371/journal.ppat.1002735 (PMC3375299; doi:10.1371/journal.ppat.1002735)
Supplement: Table S1 — Closest sequenced relatives identified, through BLAST analysis of the high-throughput sequence data, in the colonies under study, in October. Only taxa that were present in at least two colonies of either group of hives are reported. The number of hits and the average percentage identity are reported for each taxon. (PDF) [file ppat.1002735.s006.pdf]

Table S1. Closest sequenced relatives identified in the colonies under study.

taxon	LIC1	LIC2	LIC3	LIC4	LIC5	LIC6	HIC1	HIC2	HIC3	HIC4	HIC5
	low infested colonies						highly infested colonies				
	hits/id%						hits/id%				
Bifidobacterium sp.	-	-	-	-	-	-	3/85	-	-	-	2/95
Lactobacillus sp.	2/61	3/89	2/75	-	-	2/91	6/78	2/80	5/71	2/82	4/90
Leishmania sp.	-	-	-	-	-	-	-	-	-	306/84	178/86
Nosema sp.	-	9/73	-	-	9/73	9/73	10/71	9/69	8/65	-	-
Nosema ceranae	-	1465/98	-	-	1276/98	1206/99	1579/99	1794/99	1860/99	-	-
Varroa destructor	5/88	-	7/85	-	-	-	-	10/87	7/88	-	7/87
