# Supplementary material for: Synergistic Parasite-Pathogen Interactions Mediated by Host Immunity Can Drive the Collapse of Honeybee Colonies
Source: PLoS Pathog. 2012 Jun 14;8(6):e1002735. doi: 10.1371/journal.ppat.1002735 (PMC3375299; doi:10.1371/journal.ppat.1002735)
Supplement: Table S2 — Relative expression of honeybee immune genes in highly infested colonies. For each highly infested colony (HIC1 to HIC5), the RPKM ratio between that colony and the average of low infested colonies is reported; red, pink, yellow and green are used to denote genes whose ratio was lower than 0.5, between 0.5 and 0.9, between 1.1 and 2, higher than 2, respectively. Gene list from [38]; only genes with no-zero reads in at least one colony of each group are reported. Most genes were down-regulated in highly infested colonies, the most marked effect was noted for members of the Toll pathway and Serine Proteases. (PDF) [file ppat.1002735.s007.pdf]

Table S2. Relative expression of honeybee immune genes in highly infested colonies.

Feature ID	Gene name	Gene family/ pathway	Average RPKM LIC ± st. dev.	HIC1	HIC2	HIC3	HIC4	HIC5
GB18323	Abaecin	AMP	4094.42 ± 1422.35	0.67	3.63	3.06	1.70	0.94
GB17782	Apidaecin		76.12 ± 17.92	0.70	8.94	5.57	3.64	1.85
GB19468	Apisimin		18052.95 ± 11001.49	4.81	2.30	1.09	3.44	0.39
GB10036	Defensin-2		0.33 ± 0.37	0.00	51.32	12.45	0.00	4.39
GB17538	Hymenoptaecin		697.70 ± 429.50	0.46	15.63	23.47	3.66	0.93
GB16711	Hemolectin	Cellular response	8.26 ± 1.35	0.98	1.11	0.85	0.71	0.63
GB20003	Hemomucin		168.70 ± 33.33	0.82	0.93	1.02	0.76	0.93
GB11717	CTL1	C-lectin domain	5.10 ± 0.92	0.80	1.08	1.04	0.91	1.49
GB11810	CTL10		14.71 ± 1.55	1.24	1.20	1.09	0.86	0.83
GB19013	CTL11		1.79 ± 1.05	0.67	0.52	0.34	0.35	0.69
GB13808	CTL12		4.77 ± 1.14	0.52	1.00	0.97	0.77	1.06
GB14265	CTL2		53.37 ± 8.97	0.48	0.88	2.22	0.73	0.97
GB18049	CTL3		1.89 ± 0.80	0.70	0.09	0.44	0.09	0.82
GB20122	CTL4		4.56 ± 2.12	0.80	0.48	0.83	0.79	1.33
GB17330	CTL5		5.53 ± 1.92	0.34	0.54	0.74	0.91	0.88
GB11792	CTL6		12.81 ± 1.92	1.17	0.90	1.00	0.77	0.97
GB14975	CTL7		89.23 ± 22.83	1.12	0.77	0.75	0.72	0.48
GB14382	CTL8		25.59 ± 2.81	0.75	0.75	0.82	0.70	0.70
GB15050	CTL9		5.04 ± 1.04	0.35	0.85	1.12	1.03	0.43
GB14645	Eater-like	EGF Family	14.64 ± 4.84	1.04	1.66	2.83	3.18	2.65
GB14962	laminin-EGF-like		47.99 ± 4.09	0.83	0.91	1.25	0.97	0.90
GB17018	Angiopoietin	Fibrinogen	4.90 ± 1.18	0.71	0.67	0.56	0.56	0.64
GB11902	Scabrous		9.91 ± 1.99	0.49	0.45	0.75	0.65	0.78
GB10026	Galectin-1	Galectin	162.84 ± 11.12	0.81	0.72	0.91	0.80	0.78
GB18324	Galectin-2		21.50 ± 1.74	0.82	0.82	0.89	0.72	0.74
GB19452	B-gluc1	GNBPs	491.72 ± 99.46	0.59	0.79	0.83	0.80	0.89
GB19961	B-gluc2		89.67 ± 21.32	1.04	2.17	2.09	1.36	0.89
GB30209	Dscam	IG superf. genes	4.21 ± 1.00	0.69	1.17	1.08	0.87	1.27
GB14642	IGFn3-1		40.74 ± 5.69	0.58	0.55	0.67	0.56	0.69
GB11807	IGFn3-10		8.88 ± 3.25	0.66	1.18	1.69	1.48	1.65
GB12490	IGFn3-11		58.53 ± 22.51	0.90	0.82	1.06	1.38	1.90
GB14520	IGFn3-12		31.03 ± 5.86	0.54	0.77	1.11	1.15	1.50
GB10469	IGFn3-13		63.79 ± 6.50	0.68	0.68	1.47	1.14	1.11
GB12933	IGFn3-14		1.28 ± 0.35	0.82	0.93	1.42	1.29	1.71
GB11918	IGFn3-16		0.18 ± 0.06	0.00	1.38	1.00	0.35	0.00
GB11358	IGFn3-2		34.09 ± 6.60	0.66	0.64	0.83	0.68	0.69
GB15987	IGFn3-3		0.93 ± 0.23	0.73	0.94	0.79	1.18	0.86
GB13261	IGFn3-5		3.02 ± 1.86	0.42	1.02	1.34	1.98	2.60
GB10912	IGFn3-6		0.77 ± 0.19	0.39	0.50	0.43	0.36	0.99
GB11846	IGFn3-7		24.17 ± 1.47	0.70	0.71	0.72	0.64	0.70
GB14317	IGFn3-8		4.76 ± 0.43	0.90	0.96	1.05	0.75	0.61
GB16060	IGFn3-9		0.39 ± 0.19	0.19	1.12	1.21	0.64	0.71

GB30331	DREDD-Casp8	IMD	1.97 ± 0.78	1.62	0.42	1.44	1.28	2.06
GB19498	dUbc13		95.49 ± 5.35	0.80	0.65	1.02	0.57	0.73
GB30399	FADD		10.60 ± 2.90	1.23	0.88	1.33	1.39	1.14
GB17106	IKKγ-kenny		75.26 ± 14.60	0.91	0.94	1.10	0.93	0.95
GB15273	IKK-ird5		28.14 ± 4.94	0.79	0.59	0.93	0.58	0.74
GB18606	Imd		43.07 ± 5.39	1.00	1.35	1.39	1.26	1.10
GB11057	lap2		35.78 ± 8.22	0.82	1.01	1.25	0.81	0.85
GB13742	Relish		2.70 ± 1.89	0.33	2.53	1.43	0.43	0.12
GB14664	Tak1		32.92 ± 3.47	0.70	0.67	0.80	0.77	0.84
GB12159	Domeless	JakSTAT	22.81 ± 4.14	0.68	1.09	1.35	0.73	0.60
GB18362	D-PIAS		23.64 ± 4.74	0.97	0.73	1.01	0.75	0.84
GB18949	SOCS		89.91 ± 10.51	0.70	0.72	0.85	0.74	0.76
GB18923	STAT92E		32.98 ± 2.34	0.82	0.77	1.00	0.75	0.68
GB17167	Hem	JNK	15.95 ± 2.04	0.59	0.63	0.90	0.57	0.68
GB12004	Jra		25.13 ± 5.53	0.69	0.78	1.31	0.80	0.50
GB19901	puckered		51.09 ± 13.61	0.56	1.12	2.45	1.57	1.44
GB10231	Lys-1	Lysozyme	1056.72 ± 138.79	1.19	1.37	1.76	1.70	0.90
GB15106	Lys-2		673.48 ± 110.06	0.84	0.97	1.09	1.12	1.22
GB19988	Lys-3		379.90 ± 23.72	1.41	1.31	0.92	1.22	0.88
GB16401	MAPK(basket)	MAPK	31.54 ± 6.66	1.05	1.13	1.66	1.18	1.01
GB13522	MAPKKK9		27.91 ± 3.00	0.69	0.71	0.94	0.59	0.82
GB11320	RIP1		14.08 ± 1.83	1.21	1.33	1.20	1.31	1.83
GB15645	NFAT	NFAT	23.34 ± 7.12	0.52	0.87	1.20	1.30	1.45
GB12883	NimA	Phagocytosis	1.50 ± 0.69	0.65	0.57	1.36	0.90	1.21
GB14645	NimC1		14.64 ± 4.84	1.04	1.66	2.83	3.18	2.65
GB12454	NimB		20.31 ± 6.07	0.98	1.11	2.74	3.98	3.44
GB13979	NimC2		13.93 ± 5.48	1.80	3.35	1.72	3.15	1.65
GB14962	Draper		47.99 ± 4.09	0.83	0.91	1.25	0.97	0.90
GB15371	PGRP-S1	PGRP	562.70 ± 104.22	0.96	1.05	0.89	1.29	0.94
GB19301	PGRP-S2		226.29 ± 52.86	1.08	8.59	8.43	2.25	1.33
GB17879	PGRP-S3		386.93 ± 96.51	1.11	1.22	1.02	0.90	1.12
GB18313	PPO	PPO	72.79 ± 16.51	0.98	0.66	0.53	0.71	0.71
GB18767	PPOAct		69.17 ± 17.29	0.89	1.12	0.90	1.48	1.54
GB11373	Rac	RAC1 protein	230.71 ± 27.37	0.75	0.67	0.91	0.83	0.71
GB13360	lox2-like	Scav. Receptor A	8.06 ± 2.83	1.51	1.57	1.34	1.44	1.36
GB10506	AmSCR-B1	Scav. Receptor B	1.88 ± 0.68	1.49	0.24	1.01	0.82	0.66
GB11743	AmSCR-B2		6.06 ± 1.92	0.92	0.94	0.44	1.11	0.78
GB12378	AmSCR-B3		52.23 ± 0.72	0.88	0.71	1.01	0.87	0.86
GB12830	AmSCR-B4		22.68 ± 4.65	1.04	1.12	1.95	0.82	0.77
GB13813	AmSCR-B5		24.11 ± 5.32	0.67	0.31	0.67	0.63	0.54
GB14314	AmSCR-B6		51.09 ± 11.04	0.74	0.98	1.23	1.45	1.59
GB15549	AmSCR-B7		16.66 ± 4.53	0.74	0.46	0.43	0.55	0.66
GB16388	AmSCR-B8		32.51 ± 7.86	1.14	1.13	0.74	0.75	0.92
GB19925	AmSCR-C	Scav. Receptor C	38.66 ± 11.25	0.83	0.94	0.73	1.32	1.38

GB16147	cSP1		102.44 ± 13.56	1.08	1.32	1.75	1.62	0.91
GB14044	cSP14		36.28 ± 3.58	0.86	1.11	2.08	1.30	1.03
GB14247	cSP2		639.15 ± 104.10	1.11	1.47	1.48	1.63	0.83
GB16220	cSP21		8.29 ± 2.26	0.37	0.49	0.85	0.50	1.22
GB19719	cSP25		0.39 ± 0.20	0.57	1.46	1.65	1.99	1.66
GB18450	cSP26		0.36 ± 0.14	0.29	0.38	1.83	1.16	1.40
GB11698	cSP3		738.36 ± 44.10	0.91	0.88	0.86	1.15	1.14
GB14309	cSP33		0.13 ± 0.09	0.00	0.56	0.54	0.00	0.00
GB14077	cSP6		0.23 ± 0.06	0.49	1.56	0.30	1.60	1.60
GB17145	cSP7		1.48 ± 1.17	1.06	1.06	0.63	1.09	1.11
GB14366	cSPH39		3.28 ± 0.42	0.72	0.75	0.61	0.75	1.05
GB11298	cSPH42		1373.25 ± 170.28	0.83	1.21	1.37	1.13	1.05
GB14001	cSPH50		59.84 ± 8.68	1.09	0.97	1.11	1.47	1.00
GB15254	cSPH55		2.46 ± 0.94	1.02	0.44	1.18	1.66	1.12
GB14654	SP11		5.99 ± 1.80	2.31	2.26	1.26	2.41	1.49
GB19856	SP12		7.40 ± 1.60	0.70	0.92	0.89	0.71	1.04
GB15640	SP13		90.24 ± 17.24	0.86	0.95	0.96	0.89	0.97
GB18178	SP15		1.97 ± 0.50	0.66	0.85	1.87	1.42	0.84
GB14603	SP17		3500.37 ± 541.87	1.22	1.32	1.54	1.62	1.19
GB10222	SP18		127.24 ± 45.87	1.08	1.28	2.37	1.23	1.41
GB19590	SP20		2.33 ± 0.38	0.78	0.85	0.84	0.83	1.65
GB13791	SP22		3960.69 ± 678.35	1.39	1.49	1.12	1.34	1.02
GB14233	SP24		11.29 ± 2.30	1.11	1.08	0.88	1.12	1.32
GB11588	SP27	Serine proteases	30.23 ± 14.82	3.95	6.16	3.08	17.22	0.72
GB14644	SP29		4.22 ± 1.86	0.48	0.47	0.23	0.97	0.89
GB11511	SP32		6.14 ± 2.34	0.39	1.04	1.33	0.72	1.20
GB11552	SP34		17.05 ± 16.70	0.04	0.03	0.04	0.21	1.21
GB16021	SP35		604.01 ± 338.92	0.76	1.64	2.81	1.35	1.00
GB19846	SP36		12697.01 ± 1876.48	0.75	0.85	0.97	1.10	0.83
GB16214	SP38		11.41 ± 2.49	1.07	1.38	1.20	1.38	1.20
GB10646	SP4		1.68 ± 0.55	1.57	2.10	1.70	1.71	0.80
GB13263	SP40		343.19 ± 123.57	1.43	0.61	0.68	0.98	0.75
GB15453	SP44		577.04 ± 38.36	0.91	1.30	1.24	1.36	1.13
GB17654	SP45		4.86 ± 1.49	0.70	0.90	0.56	0.77	0.88
GB16367	SP46		6.07 ± 1.53	0.54	0.62	0.91	0.77	0.91
GB14774	SP47		0.12 ± 0.17	3.69	0.00	4.61	4.87	12.20
GB12379	SP48		0.67 ± 0.23	1.22	0.52	2.28	1.60	0.00
GB15317	SP49		70.54 ± 11.47	1.22	1.43	1.59	0.86	0.78
GB12300	SP5		0.72 ± 0.30	0.88	0.76	0.73	1.16	1.29
GB18767	SP8		69.17 ± 17.29	0.89	1.12	0.90	1.48	1.54
GB17345	SPH19		1.81 ± 0.37	1.28	2.05	5.17	0.82	1.10
GB18944	SPH37		8.30 ± 1.81	0.63	1.23	0.78	1.15	0.67
GB13397	SPH51		1.67 ± 0.39	0.28	0.63	0.35	0.28	0.57
GB19292	SPH52		1.80 ± 1.09	0.57	0.73	2.47	1.49	3.32
GB15702	SPH53		6.54 ± 1.76	0.78	0.86	0.95	0.88	1.17
GB15980	SPH54		18.49 ± 2.83	0.96	0.86	0.83	0.93	1.19
GB13019	SPH56		30.56 ± 2.97	0.72	0.82	1.44	0.78	1.13
GB16038	SPH57		8.94 ± 1.88	0.54	1.00	2.25	1.13	1.70

GB12605	TEP7		158.93 ± 29.22	0.72	1.01	1.11	0.80	0.75
GB18789	TEPA	TEP	9.41 ± 0.88	0.62	0.58	0.91	0.52	0.58
GB11563	TEPB		42.68 ± 6.93	0.65	0.85	1.48	0.81	0.66
GB15177	18-w		5.24 ± 2.56	1.02	1.56	2.05	2.21	1.02
GB10655	cact-1		23.26 ± 2.91	0.88	1.42	2.64	1.05	0.59
GB13520	cact-2		47.80 ± 8.40	0.73	0.98	1.40	0.87	0.69
GB19066	dorsal-1A		0.76 ± 0.40	0.17	0.00	0.64	0.00	0.00
GB18032	Dorsal-2		3.54 ± 1.02	0.66	0.67	0.45	0.18	0.58
GB14720	Mik2		12.62 ± 2.68	0.93	0.82	0.94	0.67	0.78
GB12344	MyD88		27.84 ± 5.65	1.20	1.32	1.47	0.88	0.91
GB16472	NEC LIKE		215.14 ± 45.76	0.81	1.82	1.64	0.90	0.68
GB17012	NEC LIKE		15.60 ± 3.45	0.95	0.80	0.98	0.77	1.05
GB19582	NEC LIKE		525.58 ± 62.75	0.68	0.85	1.61	1.00	0.89
GB16397	Pelle		27.86 ± 4.92	0.98	0.87	1.33	0.79	0.78
GB16970	Pellino	Toll/TLR	90.97 ± 5.17	0.74	0.72	0.90	0.87	1.00
GB14044	PSH LIKE		36.28 ± 3.58	0.86	1.11	2.08	1.30	1.03
GB15640	PSH LIKE		90.24 ± 17.24	0.86	0.95	0.96	0.89	0.97
GB13503	SPZ		16.86 ± 4.25	1.22	0.90	0.90	0.82	0.98
GB15688	SPZ		79.77 ± 16.06	0.64	0.90	0.88	0.63	0.80
GB18520	Toll (TLR)		23.06 ± 2.92	0.61	0.97	1.55	0.63	0.71
GB16299	Toll-10		0.82 ± 0.53	0.55	0.42	1.42	2.05	1.88
GB17781	Toll-6		1.15 ± 0.54	1.19	0.90	1.96	0.92	1.61
GB10640	Toll-8/Trex		0.09 ± 0.08	2.46	0.79	0.77	0.81	2.71
GB17961	Tollip		133.85 ± 14.95	0.81	1.04	1.02	0.81	0.91
GB10539	TRAF2		7.18 ± 2.26	1.69	1.03	0.99	0.94	0.68
GB15684	Tube		51.91 ± 6.75	0.98	0.74	0.98	0.68	0.91
