# Supplementary material for: Synergistic Parasite-Pathogen Interactions Mediated by Host Immunity Can Drive the Collapse of Honeybee Colonies
Source: PLoS Pathog. 2012 Jun 14;8(6):e1002735. doi: 10.1371/journal.ppat.1002735 (PMC3375299; doi:10.1371/journal.ppat.1002735)
Supplement: Table S3 — Primer pairs used for conventional RT-PCR or Quantitative Real-Time RT-PCR analyses. (PDF) [file ppat.1002735.s008.pdf]

Table S3. Primer pairs used for conventional RT-PCR or Quantitative Real-Time RT-PCR analyses.

Target	Forward and reverse primer sequences (5'-3')[a]	Expected product size (bp)
Conventional RT-PCR		
ABPV, AF150629	(F) TTATGTGTCCAGAGACTGTATCCA<br>(R) GCTCCTATTGCTCGGTTTTTCGGT	900
BQCV, NC003784	(F) TGGTCAGCTCCCACTACCTTAAAC<br>(R) GCAACAAGAAGAAACGTAAACCAC	700
CBPV, NC010711	(F) AGTTGTCATGGTTAACAGGATACGAG<br>(R) TCTAATCTTAGCACGAAAGCCGAG	455
DWV, NC004830	(F) ATCAGCGCTTAGTGGAGGAA<br>(R) TCGACAATTTTCGGACATCA	702
IAPV, NC009025	(F) GCGGAGAATATAAGGCTCAG<br>(R) TTGCAAGATAAGAAAGGGGG	586
KBV, NC004807	(F) GATGAACGTCGACCTATTGA<br>(R) TGTGGGTTGGCTATGAGTCA	415
SBV, NC002066	(F) GCTGAGGTAGGATCTTTGCGT<br>(R) TCATCATCTTCACCATCCGA	824
Nosema apis, U97150	(F) CCATTGCCGGATAAGAGAGT<br>(R) CCACCAAAAACTCCCAAGAG	269
Nosema ceranae, DQ486027	(F) CGGATAAAAGAGTCCGTTACC<br>(R) TGAGCAGGGTTCTAGGGAT	250
Quantitative Real-time RT-PCR		
DWV	(F) GCGCTTAGTGGAGGAAATGAA<br>(R) GCACCTACGCGATGTAAATCTG	69
dorsal-1A Toll/TLR	(F) TCGGATGGTGCTACGAGCGA<br>(R) AGCATGCTTCTCAGCTTCTGCCT	153
cSP33 ser proteases	(F) CGTCGGTGGTAAAGCGGCGA<br>(R) AACGGCGACCAACGTTGCCA	175
SPH51 ser proteases	(F) TGGCAATTGTCTTTGCGGGCG<br>(R) TACTTCCGCCGCCGTTACGC	196
Eater like EGE Family	(F) GGCGAGTGCACCGGCTTGAA<br>(R) GCGCCATCGCGTCATAGCCA	169
NimC2Phagocytosis	(F) GCGTGGAGGACGGGAAACCG<br>(R) ACATCGATGGCAGAGCGGCG	185
PGRP-S2	(F) GGCCACACACCAAATGCAGCAG<br>(R) CGAGGACCAGTGTGGCCATGT	177

[a] F, forward - R, reverse
